# The association between PM_2.5_ level and respiratory tract infections among children: A cross-sectional study

**DOI:** 10.3934/publichealth.2025055

**Published:** 2025-11-18

**Authors:** Hari Krismanuel, Purnamawati Tjhin

**Affiliations:** Faculty of Medicine, Universitas Trisakti, Jakarta, Indonesia

**Keywords:** air pollution, children, cross-sectional study, PM_2.5_, respiratory tract infection

## Abstract

**Background:**

PM_2.5_ is a key air pollutant that contributes to respiratory morbidity, especially in children. In Jakarta, Indonesia, PM_2.5_ levels often exceed safe thresholds. This study contributes local evidence from Indonesia, where research on the health effects of PM_2.5_ in children remains limited. To address this gap in the existing literature, particularly within the Indonesian context, this study offers novel insights by specifically investigating the association between ambient PM_2.5_ exposure and respiratory tract infections (RTIs) in school-aged children and further exploring this association within male and female subgroups, an aspect that has received limited attention in this setting.

**Objective:**

This study aims to assess the association between ambient PM_2.5_ exposure and RTIs in school-aged children, and to explore this association within male and female subgroups.

**Methods:**

This cross-sectional study was conducted among 107 children aged 6–12 years from two elementary schools: one in Jakarta (high PM_2.5_ exposure) and one in Bandung (low PM_2.5_ exposure). Data on PM_2.5_ levels were obtained from local air quality monitoring. RTI symptoms were assessed through structured interviews and physical examination. Participants were selected using random sampling. Chi-square tests and effect size calculations (phi coefficient) were used to compare groups. Potential confounders such as age, gender, and household smoke exposure were minimized through inclusion/exclusion criteria and the selection of demographically and environmentally similar school communities. Multiple binary logistic regression adjusting for confounders was also performed to assess the independent association between PM_2.5_ exposure and RTIs.

**Results:**

The Chi-square test indicated a significant association between PM_2.5_ levels and the occurrence of RTI (χ² = 22.154, df = 1, p < 0.001, φ = 0.475). Given the potential low expected counts in some cells, the statistical significance was further evaluated using Fisher's Exact Test, which also showed a significant association (p < 0.001). The prevalence of RTI was significantly higher in the high exposure group (71.43%) compared to the low exposure group (25.86%) (p < 0.001). Further analysis did not reveal significant differences in the proportion of each age group between the high and low PM_2.5_ exposure groups [χ²(1) = 0.093, p = 0.761]. Similarly, no significant differences were found in the proportion of gender between the high and low PM_2.5_ exposure groups [χ²(1) = 1.611, p = 0.204] in the total sample. Likewise, there were no significant differences in the proportion of RTI across different age groups [χ²(6) = 5.327, p = 0.503] or between genders [χ²(1) = 0.008, p = 0.928] in the total sample. However, further analysis examining the association between PM_2.5_ exposure and RTI within gender subgroups revealed a significant association in both male [χ²(1) = 10.873, p = 0.001] and female [χ²(1) = 11.755, p = 0.001] children. The estimated prevalence ratio (PR) was 2.76 (95% CI: 1.68–4.54), indicating that children in the high PM_2.5_ exposure area had approximately 2.76 times higher prevalence of RTI compared to those in the low exposure area. The absolute prevalence difference (PD) was 45.57% (95% CI: 25.9%–65.2%). Binary logistic regression analysis showed that children in the high PM_2.5_ exposure group had significantly higher odds of having RTI (OR = 7.167, 95% CI: 3.050–16.837, p < 0.001). Further analysis examining the association between maternal socioeconomic factors and both PM_2.5_ exposure and RTI occurrence revealed no statistically significant relationships. Chi-square tests showed no significant association between maternal education level (low vs. medium) and PM_2.5_ exposure group [χ²(1) = 0.045, p = 0.833], nor between maternal occupation (blue collar vs. semi-professional) and PM_2.5_ exposure group [χ²(1) = 0.006, p = 0.937]. Similarly, no significant associations were found between maternal education level and RTI [χ²(1) = 0.233, p = 0.629] or between maternal occupation and RTI [χ²(1) = 0.447, p = 0.504]. Crucially, after adjusting for potential confounders including gender, age, maternal education, and maternal occupation in a multivariate logistic regression model, the odds of having RTI remained significantly higher in children with high PM_2.5_ exposure (adjusted OR = 7.883, 95% CI: 3.228–19.250, p < 0.001).

**Conclusions:**

Children exposed to higher levels of PM_2.5_ had significantly more respiratory tract infections. These findings highlight the need for targeted public health interventions in polluted urban areas. This study is among the first to quantify this association in the Indonesian context and provides a newly developed and validated instrument (RAAEC-C instrument) for future research. These findings should be interpreted as preliminary evidence and require replication in future longitudinal studies before firm conclusions can be drawn. Further research using longitudinal designs is needed to understand the long-term impacts of PM_2.5_ exposure on children's respiratory health and to inform appropriate mitigation strategies.

## 1. Introduction

Fine particulate matter with a diameter of 2.5 µm or less (PM_2.5_) has been widely recognized as one of the most harmful air pollutants due to its ability to penetrate deep into the respiratory tract and enter the bloodstream [1]–[3]. Exposure to PM_2.5_ is associated with a range of adverse respiratory health outcomes, including airway inflammation, decreased lung function, and increased risk of respiratory tract infections and chronic respiratory diseases [1]–[5]. Research has shown that prolonged exposure to PM_2.5_ can lead to chronic respiratory conditions such as asthma and bronchitis, particularly in children whose immune systems are still developing. Children are particularly vulnerable to air pollution because their lungs and immune systems are still developing, they have higher minute ventilation per body weight, and they often spend more time outdoors than adults [6]–[9].

In Jakarta, the capital of Indonesia, the burden of air pollution is especially concerning. As one of the most densely populated and industrialized cities in Southeast Asia, Jakarta frequently experiences PM_2.5_ concentrations far exceeding the World Health Organization (WHO) air quality guideline. In August 2023, Jakarta was named the most polluted city in the world, with average PM_2.5_ concentrations exceeding safe limits by more than five times, reaching up to 80 µg/m³ on the worst days. In response, local authorities implemented remote work policies for civil servants to curb vehicle emissions and improve air quality [10]–[16]. Prolonged exposure in such environments poses significant health risks, particularly for children [6]–[9]. Furthermore, available data suggest a concerning trend of elevated PM_2.5_ levels in Jakarta over the past few years, primarily stemming from sources such as vehicle emissions, industrial activities, and biomass burning.

Research has demonstrated the adverse health effects of PM_2.5_ on children's respiratory health in various parts of the world. For example, Sugiyama et al. studied the health effects of PM_2.5_ sources on children's allergic and respiratory symptoms in Fukuoka, Japan, during the spring of 2014–2015 [17]. Another study by Xu et al. examined the acute effects of ambient PM_2.5_ on lung function among school children in Zhejiang Province, China, over the years 2014–2017 [18]. Ma et al. evaluated the association of air pollution with children's outpatient visits for respiratory diseases in the ex-heavily polluted Northwestern city, China, from 2014 to 2016 [19]. Additionally, a comprehensive literature review by Xing et al, explored the impact of PM_2.5_ on the human respiratory system, providing an in-depth analysis of epidemiological, experimental, and mechanistic studies [1]. More recently, Adhikary et al. conducted a large-scale study using PM_2.5_ data from the Atmospheric Composition Analysis Group at Washington University to assess the association between PM_2.5_ exposure and acute respiratory infections (ARI) in 223,375 children from the 2019–2021 Demographic Health Survey in India, contributing further evidence on the relationship between PM_2.5_ exposure and respiratory health outcomes in children [7].

Despite the growing body of research globally, there is a lack of studies focused on the impact of PM_2.5_ on children's respiratory health in Indonesia, particularly in urban areas like Jakarta. While studies have explored the health effects of PM_2.5_ in countries such as China, Japan, and India, evidence from Indonesia remains limited [1],[7],[17],[18]. Research is needed to understand how children in Indonesian urban environments are affected by exposure to PM_2.5_, especially considering the unique genetic, environmental, and social contexts of the region. Furthermore, genetic and racial variations may influence susceptibility to PM_2.5_-induced respiratory effects, which makes findings from other countries less generalizable to Indonesia [20]–[22]. Understanding these localized impacts is crucial for developing effective strategies to mitigate the adverse health consequences of air pollution and improve respiratory health outcomes for children in Jakarta.

Given Jakarta's alarming pollution levels and the limited availability of localized research, it is crucial to fill this gap in knowledge to better inform public health decisions. This study aims to compare respiratory health outcomes—specifically respiratory tract infections—among children exposed to high levels of PM_2.5_ in Jakarta and those living in a region with significantly lower PM_2.5_ exposure, Bandung. By comparing these two distinct regions, we aim to provide insights into the local impact of air pollution on children's respiratory health in Indonesia. Furthermore, to explore potential **variations in this association**, we also examined the association between PM_2.5_ exposure and respiratory tract infections within male and female subgroups of the study population.

This study is novel in two main ways. First, it is one of the first to empirically examine the respiratory impact of PM_2.5_ on school-age children in Indonesia, filling a crucial research gap in the Southeast Asian context. Second, it compares two distinct exposure zones—Jakarta (high PM_2.5_) and a region with significantly lower PM_2.5_ levels—allowing for a clearer understanding of the exposure-response relationship. The findings are expected to inform public health interventions, raise awareness about urban air pollution, and support evidence-based policymaking in Indonesia.

To our knowledge, this is the first study to examine the association between PM_2.5_ exposure and respiratory problems among school-aged children in Indonesia, with a specific focus on Jakarta. By comparing children from a high-exposure area (Jakarta) to those from a region with better air quality (Bandung), this study provides localized evidence on the impact of PM_2.5_ on symptoms such as coughing, wheezing, and shortness of breath.

## 2. Materials and methods

### 2.1. Research questions

What is the impact of naturally occurring differences in PM_2.5_ levels on respiratory health outcomes among children aged 6–12 years living in areas with high air pollution exposure compared to those in areas with better air quality?

### 2.2. Study design

This study employed an **analytical cross-sectional design** to evaluate the association between exposure to PM_2.5_ and the prevalence of respiratory tract infections (RTIs) in children. The population was divided into two comparison groups based on PM_2.5_ exposure levels in their residential and school environments:

- **High-exposure group**: Children living in areas with consistently high PM_2.5_ levels (poor AQI).
- **Low-exposure group**: Children living in areas with low PM_2.5_ levels (good AQI).

Unlike ecological studies that rely on group-level data, this study collected individual-level data on exposure and health outcomes, allowing for more precise estimation of associations.

The study population consisted of elementary school children aged 6–12 years attending schools located in areas with different levels of PM_2.5_.

To minimize potential confounding factors, we selected schools from communities that were as **homogeneous as possible** in terms of environmental, demographic, behavioral, and household factors (e.g., exposure to cigarette smoke, ventilation, immunization status, nutritional status, seasonality, and housing conditions). Controlled variables included:

- **Demographic factors** (age, gender, socioeconomic status).
- **Environmental factors** (exposure to cigarette smoke, population density, seasonal and weather variation).
- **Health-related factors** (immunization status, nutritional status, history of tuberculosis, congenital heart disease, cohabitation with a household member undergoing TB treatment).
- **Behavioral factors** (personal hygiene, sanitation practices, social habits).
- **Household factors** (ventilation, indoor air quality).

Homogeneity across these variables was ensured during the selection process to reduce bias and improve internal validity.

### 2.3. Study population and sampling

The study population consisted of **elementary school children aged 6–12 years** enrolled in selected schools located in areas with high and low PM_2.5_ exposure. From each exposure group (high and low PM_2.5_), one elementary school was selected. Within each selected school, a target sample of 60 students was initially recruited using stratified random sampling. Specifically, 10 students were randomly chosen from each grade level (Grade 1 to Grade 6) to ensure representation across all school years within each school.

Following the initial recruitment of 60 students from each school (totaling 120 students), participants were screened according to predetermined inclusion and exclusion criteria (see section “Eligibility Criteria”). This resulted in the exclusion of 11 students from the high PM_2.5_ exposure area school (Jakarta) and 2 students from the low PM_2.5_ exposure area school (Bandung). Consequently, the final sample size for this study comprised 107 children (49 from the high PM_2.5_ exposure area and 58 from the low PM_2.5_ exposure area).

### 2.4. Eligibility criteria

Inclusion criteria:

- Children aged 6–12 years.
- Enrolled in selected elementary schools located in areas with high or low PM_2.5_ exposure.
- Obtained written parental/guardian consent and gave verbal assent to participate.
- Present at school on the day of data collection.

Exclusion criteria:

- Children with poor nutritional status (as determined by anthropometric measurements, including height and weight to calculate BMI).
- History of congenital heart disease.
- History of pulmonary TB treatment.
- Household member currently undergoing TB treatment.
- Current smoker or exposed to active smoking at home.
- Refusal by the child or parent/ guardian to participate or to continue participation in the research.

### 2.5. Study setting and procedure

The study was conducted in two urban areas in Indonesia with contrasting levels of particulate matter (PM_2.5_) exposure: Jakarta, characterized by high PM_2.5_ levels, and Bandung Regency, known for its relatively lower PM_2.5_ levels, based on mapping data from local air quality monitoring stations. Within these urban areas, two elementary schools were strategically selected: SD Kedoya (Kedoya Elementary School) in West Jakarta, representing the high PM_2.5_ exposure site, and SD Pangalengan (Pangalengan Elementary School) in Pangalengan, Bandung Regency, representing the lower PM_2.5_ exposure site.

Data collection at SD Kedoya (Kedoya Elementary School), situated in West Jakarta, Jakarta, was carried out on May 6, 2024. Subsequently, data collection at SD Pangalengan (Pangalengan Elementary School), located in Pangalengan, Bandung Regency, West Java, took place on May 11, 2024. The data collection period across both sites spanned five days.

Data collection was carried out in several stages:

1. **Random selection of schools** in each city and eligible students aged 6–12 years from class rosters.

2. **Informed consent** obtained from parents or guardians, and verbal assent obtained from the children.

3. **Face-to-face interviews** with parents or guardians to collect data on symptoms of RTIs, including cough, wheezing, shortness of breath, and exacerbation of asthma.

4. **Application of exclusion criteria**, including screening for:

 ○Poor nutritional status;

 ○Congenital heart disease;

 ○Current or past TB treatment;

 ○Household member with active TB;

 ○Smoking behavior;

 ○Refusal to participate or continue in the study.

5. **Determination of nutritional status** by measurement and anthropometry

6. **Clinical examination** of each child's oral cavity, pharynx, nasal passages, and chest was conducted to support the diagnosis of RTIs.

### 2.6. Stages of research implementation

The implementation of this study involved several structured steps. First, air quality mapping was conducted in three locations within Jakarta and Bandung to identify areas with the highest and lowest PM_2.5_ levels. Based on this mapping, Kedoya Elementary School in Jakarta and Pangalengan Elementary School in Bandung were selected as the study sites.

Following school selection, students aged 6–12 years were randomly selected from class rosters across grades 1 to 6. Informed consent from parents and verbal assent from children were obtained prior to participation. Each child underwent an interview and a brief clinical examination to assess symptoms of respiratory tract infection, such as coughing, wheezing, and shortness of breath.

Children who met exclusion criteria—such as poor nutritional status, history of chronic illnesses, tuberculosis exposure, or parental refusal—were excluded from the study. The final dataset was then subjected to statistical analysis to examine differences in RTI prevalence between the two exposure groups.

The research was conducted according to standard procedures for cross-sectional observational studies. Throughout the implementation, we adhered to the STROBE (Strengthening the Reporting of Observational Studies in Epidemiology) guidelines to ensure transparent reporting [23],[24]. The completed STROBE checklist is provided in Table S1 of the Supplementary Materials.

### 2.7. Sample size determination

The sample size for a cross-sectional study depends on several factors, including the desired level of confidence, the margin of error, the expected prevalence of the health effects, and data variability. There are standard formulas to calculate sample size, such as the one for estimating a population proportion (used for binary outcomes like the presence or absence of health effects). The formula is as follows [25]–[27]:

$$n=\frac{{\left({\text{z}}_{\frac{\text{α}}{2}}+{\text{z}}_{\text{β}}\right)}^{2}\cdot [\text{P}1\cdot \left(1-\text{P}1\right)+\text{P}0\cdot \left(1-\text{P}0\right)]}{{\left(\text{P}1-\text{P}0\right)}^{2}}$$ (1)

Where:

- Z_α/2_ is the Z-score corresponding to the desired significance level (α/2).
- Z_β_ is the Z-score corresponding to the desired power (1−β).
- P_avg_ is the average of the expected proportions (P0 and P1).
- P0 is the expected proportion in the unexposed group.
- P1 is the expected proportion in the exposed group.
- R is the ratio of unexposed to exposed participants.

Using this formula with the assumed proportions, a significance level of 1%, and a power of 95%, the required sample size was determined to be 34 participants per group. This calculation was based on an assumed prevalence difference of approximately 40%, derived from previous studies in similar urban pediatric populations. To strengthen the study's statistical power and account for potential non-response, data loss, or misclassification, we increased the sample size beyond the minimum requirement. This larger sample size was specifically chosen to enhance our ability to detect smaller effect sizes and to allow for more nuanced analyses of potential interactions between variables. To account for potential confounding effects of other variables such as age, gender, socioeconomic status, parental smoking, history of respiratory illness, and household conditions, confounding was addressed through a controlled study design (exclusion criteria, homogenized site selection), and statistical comparisons of baseline characteristics between exposure groups. The adjusted odds ratios (aOR) and their corresponding 95% confidence intervals will be reported to assess the independent effect of PM_2.5_ exposure on RTI prevalence. A larger sample improves the precision of effect estimates, reduces the risk of Type II errors, and enhances the generalizability of findings within the target population. The sample size calculation was performed a priori.

### 2.8. Research subjects

A total of 107 children participated in the study, with 49 from areas with high PM_2.5_ exposure (poor air quality) and 58 from areas with low PM_2.5_ exposure (good air quality). Participants were selected through random sampling from school rosters, followed by screening based on predetermined eligibility and exclusion criteria.

Each subject was examined for respiratory symptoms, including cough, wheezing, shortness of breath, and aggravation of pre-existing respiratory conditions such as asthma.

### 2.9. Data collection instruments

A modified instrument, named the Respiratory Tract Infection, Asthma Exacerbation Assessment and Exclusion Criteria Checklist for Children (RAAEC-C), was developed for this study. This tool was designed to fulfill two main objectives: (1) to assess the presence of RTI symptoms and signs in children, and (2) to identify and exclude participants with known confounding factors that may independently cause RTI symptoms regardless of PM_2.5_ exposure. These include poor nutritional status, history of TB, congenital heart disease, household smoking exposure, and incomplete immunization.

The RAAEC-C was developed based on multiple validated references, including the WHO's Integrated Management of Childhood Illness (IMCI) and the ISAAC (International Study of Asthma and Allergies in Childhood) framework, and national pediatric clinical guidelines [28]–[31]. It was constructed based on established clinical criteria and expert input to ensure contextual relevance and practical applicability in school-aged populations.

The RAAEC-C form is an original copyrighted work by the author and has been formally registered for intellectual property rights (IPR). Unauthorized use, reproduction, or distribution of this tool without prior permission is prohibited.

Data was collected using the RAAEC-C structured checklist by the research team (physicians). This tool includes:

 ○**A checklist of RTI symptoms**, such as cough, wheezing, shortness of breath, and aggravation of asthma.

 ○**Eligibility and exclusion criteria**, including a history of pulmonary TB, exposure to household smoke, congenital heart disease, or current use of TB medication.

 ○**Early life health history**, including immunization status and exclusive breastfeeding for the first six months of life.

 ○**Socioeconomic background**, including mother's education level and employment status.

 ○**Anthropometric measurements and indicators**.

Anthropometric measurements, including body weight and height, were taken using standardized equipment to assess each child's nutritional status. These raw measurements were used to calculate anthropometric indicators, such as Body Mass Index (BMI), and were interpreted according to WHO growth standards. The BMI-for-age z-scores were derived to classify the children's nutritional categories (e.g., underweight, normal, overweight). This combined approach allowed for both accurate field assessment and meaningful interpretation of the children's growth and nutritional status [32]–[36].

 ○**Health assessment:** Conducted by the research team (physicians); this included **physical examination**, including vital signs, oral cavity and throat, nasal passages, heart and lung examination to assess heart problems, and signs of respiratory tract infection and exacerbation of asthma. This step followed a standardized clinical protocol to support the diagnosis of RTIs and asthma.

In this study, the RAAEC-C instrument's content validity was confirmed through an expert review, with the Content Validity Index (CVI) indicating satisfactory relevance and comprehensiveness. The instrument's reliability was then assessed by evaluating inter-rater agreement. This analysis was performed on the categorical diagnosis using **Cohen's Kappa** to confirm a high level of consistency among assessors. Additionally, the consistency of continuous measurements, such as BMI-for-age z-scores and anthropometric indicators, was confirmed with the **intraclass correlation coefficient (ICC)**.

### 2.10. Data collection

Interviews and assessments were conducted in the presence of parents or guardians to enhance the accuracy and reliability of responses. These were performed at school during school hours. The research team used the Respiratory Tract Infection, Asthma Exacerbation Assessment and Exclusion Criteria Checklist for Children (RAAEC-C) form and a standardized protocol to collect data on RTI symptoms, including cough, wheezing, and shortness of breath.

To ensure representativeness across different age groups, a **stratified random sampling** method was employed. Within each school located in both the high and low PM_2.5_ exposure areas, 10 students were randomly selected from each grade (grades 1 to 6) [37],[38]. These students were then screened for eligibility based on predefined inclusion and exclusion criteria.

Data collection was conducted through structured interviews and physical assessments. The structured questionnaire was used to obtain information related to demographic data (gender, age) and socioeconomic factors (mother's education, mother's employment status), household smoking exposure, and medical history. It also included a checklist of respiratory tract infection (RTI) symptoms such as coughing, wheezing, shortness of breath, and exacerbation of asthma.

Anthropometric measurements were taken to assess the children's nutritional status. Body weight and height were recorded using standardized equipment, and BMI-for-age z-scores were calculated based on WHO growth standards. These assessments informed both the evaluation of health status and the application of exclusion criteria related to malnutrition.

The presence of respiratory tract infection (RTI) symptoms was assessed through structured interview and clinical examination. Clinical examination was conducted to support the diagnosis of RTIs. Each child's respiratory condition was objectively assessed by trained physicians using the **clinical section** of the Respiratory Tract Infection, Asthma Exacerbation Assessment and Exclusion Criteria Checklist for Children (RAAEC-C). This included structured assessments of nasal examination, oral cavity and throat inspection (including tonsils), chest auscultation, and signs of respiratory distress (e.g., nasal flaring, chest indrawing, use of accessory muscles).

### 2.11. PM_2.5_ measurement

PM_2.5_ exposure in this study was estimated using a **mapping-based air quality survey**, a methodology that involves the strategic selection of locations to map and characterize an area's air quality levels. This method efficiently identifies locations with contrasting pollutant concentrations, allowing for the selection of high- and low-exposure sites for a study.

In both Jakarta and Bandung, three locations were initially selected in each city based on preliminary information regarding air pollution. At each of these locations, PM_2.5_ concentrations were measured using portable air quality monitors over a defined period. From the three measured locations in each city, the site with the highest PM_2.5_ concentration was selected as the high-exposure school, and the site with the lowest PM_2.5_ concentration was selected as the low-exposure school. This approach allowed for the identification of locations representing the extremes of PM_2.5_ exposure within each city.

This approach was chosen because it is practical, cost-effective, and allows rapid identification of areas with contrasting PM_2.5_ exposure levels suitable for a cross-sectional study design. **Direct personal exposure monitoring was not feasible due** to the high cost and logistical complexity of providing individual monitors for each participant, **especially across a large study area, before specific school locations and participants had been selected**. **Time-weighted exposure models** were also considered **impractical, as they require detailed data on individual activity patterns and microenvironment concentrations, which were not available for the broad areas initially considered. Other complex exposure models**, such as satellite-based estimates or fixed-site long-term monitoring, were not used because **they either lack the spatial resolution necessary** for school-level selection or **do not capture local variations in air quality relevant to the participants**. Therefore, a mapping-based air quality survey was chosen to efficiently identify specific high- and low-exposure locations before participant selection.

Overall, the mapping-based survey provided an **efficient and reliable method** to distinguish high- and low-exposure locations for the study **while maintaining feasibility** within the available resources.

### 2.12. Handling of missing data

Given the cross-sectional study design, characterized by direct interaction with participants and the implementation of standardized data collection protocols, the likelihood of missing data was anticipated to be minimal. Nevertheless, sporadic missingness could have occurred due to factors such as respondent fatigue or oversights during the data collection process. For instances of minimal missing data, a complete case analysis was employed, where participants with incomplete data for specific variables were excluded from analyses involving those variables. However, should the extent of missing data be substantial enough to potentially compromise the required minimum sample size for adequate statistical power, replacement participants were recruited from the same strata using the identical stratified random sampling methodology employed in the initial participant selection. This approach aims to maintain the intended sample size and representativeness, mitigating potential bias associated with significant data loss.

As this was a cross-sectional study, the concept of “loss to follow-up” was not applicable.

### 2.13. Efforts to address potential confounding variables

To minimize the potential for confounding variables, several strategies were implemented in the study design and participant selection. Children with a history of respiratory tract infection other than the current episode and those with a history of tuberculosis or exposure to smoking were excluded from the study. Furthermore, study sites were selected to have relatively homogenous socioeconomic backgrounds (primarily families of farmers and laborers) and similar household conditions (small houses in densely populated areas) to reduce the influence of these factors.

The distribution of key demographic variables, age and gender, was compared between the high and low PM_2.5_ exposure groups using a Chi-square test. To address potential confounding variables, baseline demographic characteristics such as age and gender were compared between exposure groups to ensure comparability prior to the main analysis. Detailed results of these comparisons are presented in the Results section.

### 2.14. Research variables

- **Independent variable**: PM_2.5_ exposure level (high vs. low).
- **Dependent variable**: Presence or absence of RTI symptoms. RTI status was assessed based on parent-reported symptoms and supported by clinical examination findings (e.g., oral cavity and chest assessment).
- **Confounding variables**: Age, gender, socioeconomic status (homogenized by selecting schools in similar socioeconomic areas), nutritional status (assessed using anthropometric measurements as described earlier), exposure to smoking (controlled through exclusion criteria), asthma, immunization status, household density, and history of pulmonary TB (controlled through exclusion criteria).

Potential confounding variables were controlled through study design (inclusion/exclusion criteria, homogenized site selection) and assessment of baseline characteristics, including statistical comparison of age and gender distribution between exposure groups (as detailed in the “*Efforts to Address Potential Confounding Variables*” section), and multivariate logistic regression.

### 2.15. Statistical analysis

Descriptive statistics were used to summarize the demographic and clinical characteristics of the study population. Differences in the prevalence of RTI symptoms between high and low PM_2.5_ exposure groups were analyzed using the Chi-square test. To explore potential gender-specific associations, stratified analyses were also conducted using separate Chi-square tests for male and female subgroups to assess the association between PM_2.5_ exposure level (high vs. low) and the occurrence of RTI within each gender. A significance level of α = 0.01 was applied. A power analysis was performed to determine the appropriate sample size, ensuring the study had 95% power to detect a significant difference in the prevalence of RTI symptoms between the high and low PM_2.5_ exposure groups, with a significance level of α = 0.01. Furthermore, binary logistic regression analysis was conducted to estimate the odds ratio (OR) for the association between PM_2.5_ exposure (as a binary variable: high vs. low) and the occurrence of RTI (as a binary outcome: yes vs. no).

In addition, **prevalence ratio (PR)** and **prevalence difference (PD)** were calculated to quantify the magnitude of the observed differences in the proportions of RTI symptoms between the exposure groups. The **PR** was calculated using the formula [39],[40]:

$$PR=\frac{\text{Prevalence in high exposure group}}{\text{Prevalence in low exposure group}}=\frac{\text{a}/(\text{a}+\text{b})}{\text{c}/(\text{c}+\text{d})}$$ (2)

where:

- a = number of children with respiratory tract infections in the high PM_2.5_ area.
- b = number of children without respiratory tract infections in the high PM_2.5_ area.
- c = number of children with respiratory tract infections in the low PM_2.5_ area.
- d = number of children without respiratory tract infections in the low PM_2.5_ area.

where risk is the proportion of individuals experiencing RTI symptoms in each exposure group. The **PD** was calculated as:

$$PD=\text{Prevalence in high exposure group}-\text{Prevalence in low exposure group}=\frac{\text{a}}{\text{a}+b}-\frac{c}{c+d}$$ (3)

Multiple binary logistic regression was conducted to estimate the association between PM_2.5_ exposure and the occurrence of RTIs, with adjustment for potential confounders including gender, age, mother's education, and mother's employment status. The results were presented as ORs with 95% confidence intervals. However, due to the relatively small sample size and limited number of outcome events, the multivariate model was interpreted with caution, as overfitting and instability could affect the results [41],[42].

In addition, to quantify the magnitude of observed differences, **Prevalence Ratio (PR)** and **Prevalence Difference (PD)** were calculated. Confounding was minimized through careful study design, strict inclusion and exclusion criteria, and the selection of homogeneous school communities [39],[40].

To further evaluate the strength of the association between PM_2.5_ exposure and RTIs, the effect size was calculated using the **Phi coefficient (φ)** [43]. The Phi coefficient measures the association between two binary variables:

- φ values close to 0 indicate no association.
- φ values close to ±1 indicate strong association.

The Phi coefficient formula for a 2 × 2 contingency table is as follows:

$$\varphi =\frac{ad-bc}{\sqrt{\left(a+b)(c+d)(a+c)(b+d\right)}}$$ (4)

where **a**, **b**, **c**, and **d** are the frequencies in the 2 × 2 contingency table representing the distribution of RTI symptoms across exposure groups. The absolute value of the Phi coefficient (|**φ**|) was interpreted using standard thresholds (e.g., |0.1| = small, |0.3| = moderate, |0.5| = large association). The sign of **φ** indicates the direction of the association.

To minimize the risk of overfitting, given the relatively small sample size, we followed the 10-events-per-variable rule when specifying logistic regression models. In addition, sensitivity analyses were conducted by fitting models with a reduced set of covariates and models including only PM_2.5_ exposure. Model diagnostics, including the Hosmer–Lemeshow test and residual plots, were performed to assess model fit and stability.

Sensitivity analyses using reduced models (including only PM_2.5_ and selected covariates) yielded similar odds ratios in both direction and magnitude, supporting the robustness of the findings. In addition, the RAAEC-C instrument used for RTI assessment underwent expert review for content validity, and its reliability was assessed using several metrics. Inter-rater agreement was evaluated with **Cohen's Kappa** for the final categorical diagnosis and the **intraclass correlation coefficient (ICC)** for continuous measurements, ensuring that outcome measurements were both consistent and credible.

All analyses were performed using SPSS version 26. The reporting of methods and results was conducted in accordance with the STROBE (Strengthening the Reporting of Observational Studies in Epidemiology) checklist for cross-sectional studies [23],[24]. This completed checklist can be found in Table S1 of the Supplementary Materials.

### 2.16. Ethical clearance

The study was conducted in accordance with ethical guidelines and was approved by the Ethical Review Committee of the Faculty of Medicine, Universitas Trisakti (ethical permission number 059/KER/FK/III/2024). The **written informed consent form**, detailing the study's objectives, procedures, potential risks, and benefits, was submitted to the Ethical Review Committee as part of the ethical approval process.

Before participation, written informed consent was obtained from parents/guardians, and verbal assent was obtained from children. The consent process included a thorough explanation of the study's objectives, procedures, potential risks, and benefits. Parents/guardians and participants were informed that their participation was voluntary and that they could withdraw at any time without consequences. The consent process was witnessed by an impartial third party to ensure its integrity, including a representative from the school and a member of the research team. Parents/guardians who could not speak Indonesian or were illiterate were excluded to ensure a fully understood consent process.

To ensure confidentiality, all data were anonymized, and datasets were securely stored and accessible only to the research team.

## 3. Results

We measured PM_2.5_ levels in three locations in Jakarta (areas with poor/unhealthy air quality) and three locations around Bandung (areas with relatively good air quality) to identify appropriate study sites. Based on the obtained data, one elementary school in Kedoya, West Jakarta (with high PM_2.5_ levels), and one elementary school in Pangalengan, Bandung Regency (with low PM_2.5_ levels), were selected. **Air quality measurements** from all six locations are presented in **Table 1**.

**Table 1.** Ambient PM_2.5_ and PM_10_ levels in study areas.

Area	Location	PM_2.5_ (µg/m³)	PM_10_ (µg/m³)
Jakarta	Kedoya	**57**	**67**
	Cilandak barat	49	56
	Mangga dua	32	37
Bandung	Pangalengan	**15**	**23**
	Leuwi Panjang	27	33
	Padalarang	30	35

A total of 55 students from Jakarta and 60 students from Pangalengan were initially enrolled through random sampling. Interviews were conducted with each child and their parents, followed by a physical examination guided by a structured questionnaire. This questionnaire assessed signs and symptoms of respiratory tract infections and asthma and also gathered information on potential confounding variables.

Children were excluded if they had poor nutritional status, incomplete immunization, chronic lung disease, a history of pulmonary tuberculosis (or cohabiting with someone who had it), congenital heart disease, long-term pulmonary treatment, or if they or their parents refused to participate. Exclusion was also carried out for children and parents who were unwilling to continue the research. After exclusions (6 students from Kedoya and 2 from Pangalengan), the final sample consisted of **49 students in Kedoya** and **58 students in Pangalengan**.

In Kedoya, there were 28 boys (57.14%) and 21 girls (42.86%), while in Pangalengan, there were 27 boys (46.55%) and 31 girls (53.45%). In the high PM_2.5_ exposure group (Kedoya), 35 students (71.43%) had respiratory tract infections, while 14 (28.57%) were healthy. In the low PM_2.5_ exposure group (Pangalengan), 15 students (25.86%) had infections, and 43 (74.14%) were healthy. Age, gender, and health status distributions for both groups are shown in **Table 2**. Notably, there were no reported cases of a history of asthma or asthma attack/exacerbation in either group.

We tested for differences in the proportions of age and gender between the high and low PM_2.5_ exposure groups. The Chi-square test for age yielded a p-value of 0.761, and for gender, a p-value of 0.928—both greater than the significance level (α = 0.01). Thus, there were no statistically significant differences in age and gender distributions between the two groups.

Furthermore, the prevalence of respiratory tract infections was significantly higher in the Kedoya group (71.43%) compared to the Pangalengan group (25.86%) (p < 0.001, based on the Chi-square test).

Since the groups were comparable in terms of demographic variables, further analysis was conducted to compare the proportion of respiratory tract infections. The Chi-square test for respiratory tract infection status showed a p-value < 0.001, indicating a statistically significant difference between the groups. Students in the high exposure area (Kedoya) had a significantly higher rate of respiratory tract infections compared to those in the low exposure area (Pangalengan).

Regarding the socioeconomic status of the parents/guardians, the majority of mothers in both Kedoya (65.31%) and Pangalengan (67.24%) had a low level of education (no school to Junior High School). A smaller proportion of mothers had a medium level of education (Senior High School/Vocational High School) in Kedoya (34.69%) and Pangalengan (32.76%). No mothers in either group had a high level of education (Diploma to Doctoral Degree).

A summary of the Pearson Chi-square test results examining the associations between age, gender, socioeconomic status, PM_2.5_ exposure, and respiratory tract infections in the two schools is presented in **Table 3**.

**Table 2.** Student demographics, health status, and socioeconomic status of parents (guardians).

Characteristics	Sub groups	SD in Kedoya	SD in Pangalengan
		n (%)	n (%)
Age	6	0 (0%)	1 (1.72%)
	7	0 (0%)	8 (13.79%)
	8	8 (16.33%)	6 (10.34%)
	9	12 (24.49%)	7 (12.07%)
	10	6 (12.24%)	12 (20.69%)
	11	9 (18.37%)	10 (17.24%)
	12	14 (28.57%)	14 (24.14%)
Gender	Boy	28 (57.14%)	26 (44.83%)
	Girl	21 (42.86%)	32 (55.17%)
Respiratory tract	Yes	35 (71.43 %)	15 (25.86%)
Infections	No	14 (28.57%)	43 (74.14%)
History of asthma	No	0 (0%)	0 (0%)
Asthma attack/exacerbation	No	0 (0%)	0 (0%)
Socioeconomic status of parents/ guardians			
1. Mother's education	a. Low (No school to Junior High School).	32 (65.31%)	39 (67.24%)
	b. Medium (Senior High School/ Vocational High School.	17 (34.69%)	19 (32.76%)
	c. High (Diploma to Doctoral Degree).	0 (0%)	0 (0%)
2. Mother's occupation	a. Blue collar/ manual/informal	40 (81.63%)	48 (82.76%)
	b. Semi-professional/Technician	9 (18.37%)	10 (17.24%)
	c. Professional/Managerial	0 (0%)	0 (0%)

**Table 3.** Pearson Chi-square test results: age, gender, socioeconomic status, PM_2.5_ exposure, and respiratory tract infections.

Statistical analysis	Groups/Subgroups	Crosstab	Value	df	Asym. Sig. (2 sided)
Pearson Chi-square of independence	Total	Age × PM_2.5_	0.093	1	0.761
	Total	Gender × PM_2.5_	1.611	1	0.204
	Total	PM_2.5_ × RTI	22.154	1	<0.001
	Total	Age*RTI	5.327	6	0.503
	Total	Gender × RTI	0.008	1	0.928
	Male	PM_2.5_ × RTI (male)	10.873	1	0.001
	Female	PM_2.5_ × RTI (female)	11.755	1	0.001
	Total	Mother's education × PM_2.5_	0.045	1	0.833
	Total	Mother's occupation × PM_2.5_	0.006	1	0.937
	Total	Mother's education × RTI	0.233	1	0.629
	Total	Mother's occupation × RTI	0.447	1	0.504

Note: Level of significance (α) = 0.01, research power = 95%. Asym. Sig. = Asymptotic significance (two-sided p-value).

Further examination of the socioeconomic characteristics of the study population revealed that the majority of mothers in both the high and low PM_2.5_ exposure groups had a low level of education. To assess whether the mother's education level was associated with the PM_2.5_ exposure group, a Chi-square test was conducted. The results showed no statistically significant association between maternal education level (low vs. medium) and PM_2.5_ exposure group (low vs. high) [χ²(1) = 0.045, p = 0.833]. This suggests that in this study, maternal education level was not significantly different between the two PM_2.5_ exposure groups.

The Chi-square test results [Pearson Chi-square value = 0.006, df = 1, asymptotic significance (2-sided) = 0.937] indicated that there is no statistically significant association between the mother's occupation (blue collar vs. semi-professional) and the PM_2.5_ exposure group (low vs. high) in this sample (p = 0.937, which is greater than the conventional significance level of 0.05).

Looking at the crosstabulation:

- Among mothers with blue-collar occupations, 54.0% of their children are in the low PM_2.5_ exposure group, and 46.0% are in the high PM_2.5_ exposure group.
- Among mothers with semi-professional occupations, 55.0% of their children are in the low PM_2.5_ exposure group, and 45.0% are in the high PM_2.5_ exposure group.

The proportions of children in each PM_2.5_ exposure group were very similar across the two maternal occupation categories.

To examine the potential association between maternal education level and the occurrence of RTIs, a Chi-square test was conducted. The results showed no statistically significant association between maternal education level (low vs. medium) and RTI [χ²(1) = 0.233, p = 0.629].

Similarly, the association between mother's occupation (blue collar vs. semi-professional) and the occurrence of RTIs was examined using a Chi-square test. The results showed no statistically significant association between mother's occupation and RTI [χ²(1) = 0.447, p = 0.504].

The relationship between PM_2.5_ exposure level and the occurrence of RTIs was examined using a cross-sectional study design. Statistical analyses included a Chi-square test of independence, prevalence ratio, prevalence difference, Phi coefficient to assess the strength of association, and binary logistic regression to estimate the odds ratio (OR).

Chi-square and Fisher's Exact Test for PM_2.5_ and RTIs:

As shown in Table 3, the Chi-square test indicated no statistically significant differences in the proportions of age (p = 0.761) and gender (p = 0.204) between the Kedoya and Pangalengan groups. This suggests that the two study groups were relatively comparable in terms of age and gender distribution.

The Chi-square test indicated a significant association between PM_2.5_ levels and the occurrence of RTI (χ² = 22.154, df = 1, p < 0.001). Given that some cells had low expected counts, the statistical significance was also evaluated using Fisher's Exact Test, which also showed a significant result (p < 0.001).

A Chi-square test of independence was conducted to examine the association between gender (male vs. female) and the occurrence of RTIs in the study population (N = 107). The results of the analysis (Pearson Chi-square = 0.008, df = 1, p = 0.928) indicate **no statistically significant association** between gender and the presence of RTIs. As shown in the crosstabulation, the proportion of males experiencing RTI (46.3%) was similar to that of females (47.2%). This suggests that, in this sample, gender was not a significant factor in predicting the likelihood of having an RTI when considering the entire study group.

The complete SPSS output, including the case processing summary, gender × RTI crosstabulation, and the Chi-square tests table, supporting the analysis of the association between gender and RTIs, can be found in Table S2 of the Supplementary Materials.

Subgroup analysis was then conducted to examine the association between PM_2.5_ exposure level and RTI incidence, specifically within the male population (n = 54). The Chi-square test revealed a statistically significant association between PM_2.5_ exposure and RTI [χ²(1) = 10.873, p = 0.001]. As shown in the crosstabulation, a higher proportion of males in the high PM_2.5_ exposure group experienced RTI (67.9%) compared to those in the low PM_2.5_ exposure group (23.1%). This suggests that among males in this study, higher exposure to PM_2.5_ was significantly associated with an increased likelihood of developing RTIs.

The complete SPSS output for the subgroup analysis in males, including the case processing summary, PM_2.5_ × RTI crosstabulation, and the Chi-square tests table, can be found in Table S2 of the Supplementary Materials.

A similar subgroup analysis was performed for the female population (n = 53) to examine the association between PM_2.5_ exposure level and RTI incidence. The Chi-square test also revealed a statistically significant association between PM_2.5_ exposure and RTI [χ²(1) = 11.755, p < 0.001]. Consistent with the findings for males, the crosstabulation showed a higher proportion of females in the high PM_2.5_ exposure group experiencing RTIs (76.2%) compared to those in the low PM_2.5_ exposure group (28.1%). This indicates that among females in this study, higher exposure to PM_2.5_ was also significantly associated with an increased likelihood of developing RTIs.

The complete SPSS output for the subgroup analysis in females, including the case processing summary, PM_2.5_ × RTI crosstabulation, and the Chi-square tests table, can be found in Table S2 of the Supplementary Materials.

### 3.1. Prevalence ratio (estimated from crosstabs)

Based on the crosstabulation table, the prevalence of RTIs in the high PM_2.5_ exposure group was 71.4%, while in the low PM_2.5_ exposure group it was 25.9%. The estimated prevalence ratio (PR) was calculated as:

PR = 2.7567 ≈ 2.76

This suggests that the prevalence of RTI was approximately 2.76 times higher in the high PM_2.5_ exposure group compared to the low PM_2.5_ exposure group.

### 3.2. Prevalence difference (PD)

The absolute PD between the two groups was:

PD = 71.4% − 25.9% = 45.57%

This indicates that the prevalence of RTI was 45.57% higher in the high PM_2.5_ exposure group compared to the low PM_2.5_ exposure group.

### 3.3. Odds ratio (OR) from logistic regression

Binary logistic regression analysis was conducted to further examine the association between PM_2.5_ exposure and RTI. Consistent results were obtained across different analytical methods. The OR for RTI in the high PM_2.5_ exposure group (compared to the low exposure group) was found to be 7.167 (95% CI: 3.050–16.837) across logistic regression (using simple contrast), risk estimate, and Mantel–Haenszel analyses. This OR was statistically significant (p < 0.001).

### 3.4. Note on the interpretation of odds ratio

Given the prevalence of RTI in this study (25.9% in the low exposure group and 71.4% in the high exposure group), the OR may overestimate the magnitude of the association compared to the prevalence ratio, especially when the outcome is not rare, as is the case with RTI in this study. However, the consistent OR of 7.167 obtained from multiple robust methods strengthens the evidence of a substantial association between high PM_2.5_ exposure and increased odds of RTI. While theoretically distinct, the consistency across these measures provides a more comprehensive understanding of the relationship in this specific dataset.

### 3.5. Strength of association (Phi coefficient)

The strength of the association between PM_2.5_ exposure level and the occurrence of respiratory tract infections was assessed using the Phi coefficient (**φ** = 0.475). This value indicates a **moderate to large association** between PM_2.5_ exposure levels and the occurrence of respiratory tract infections. The association was statistically significant (p < 0.001). This further supports the conclusion that students in high-exposure areas experienced significantly more respiratory issues compared to those in low-exposure areas.

**Table 4** provides a consolidated summary of the association measures between PM_2.5_ exposure and the prevalence of RTIs in children, including the prevalence ratio, prevalence difference, odds ratio, and Phi coefficient derived from the statistical analyses.

**Table 4.** Summary of associations between PM_2.5_ exposure and RTI outcomes in children.

Measure of Association	Value	Interpretation
Prevalence ratio	2.76 (95% CI: 1.68–4.54)	Children exposed to high PM_2.5_ levels had 2.76 times higher prevalence RTIs compared to those with low exposure.
Prevalence dependence	45.57% (95% CI: 25.9%–65.2%)	The prevalence of RTIs was 45.57% higher in the high PM_2.5_ exposure group compared to the low exposure group.
Odds ratio from logistic regression	7.167 (95% CI: 3.050–16.837)	Children in the high exposure group had 7.167 times higher odds of having RTIs, with a statistically significant association.
Phi coefficient	0.475	Indicates a moderate to strong positive association between PM_2.5_ exposure and RTIs symptoms.

### 3.6. Sensitivity analyses

Sensitivity analyses were conducted to evaluate the robustness of the observed association between high PM_2.5_ exposure and RTIs. These results indicate that the association between high PM_2.5_ exposure and RTIs is **robust and not substantially affected** by the inclusion of additional covariates (**Table 5)**.

The results of our sensitivity analyses, as presented in Table 5, further support the robustness of the observed association. Across the different models, the OR estimates for the association between high PM_2.5_ exposure and RTIs ranged from 7.167 to 7.883, with all corresponding 95% confidence intervals consistently remaining above 1.0. This indicates that our primary finding is stable and not substantially influenced by the inclusion of additional covariates.

**Table 5.** Sensitivity analyses of the association between PM_2.5_ exposure and RTIs in children.

Model	OR (PM_2.5_ high vs. low)	95% CI
Unadjusted (PM_2.5_ only)	7.167	3.050–16.837
Partially adjusted	7.578	3.159–18.182
Fully adjusted	7.883	3.228–19.250

### 3.7. Multiple binary logistic regression analysis

Following the association summary, a multiple binary logistic regression analysis was conducted to evaluate the independent effect of PM_2.5_ exposure on the occurrence of RTIs after adjusting for potential confounding variables, including gender, age, mother's education level, and mother's employment status. The results are presented in **Table 6**.

**Table 6.** Multiple binary logistic regression analysis for confounding factors associated with RTIs among children.

Predictor variable	Regression coefficient (B)	Standard Error (S.E.)	Odds Ratio [Exp(B)]	95% Confidence Interval [Exp(B)]	p-value (Sig.)
PM_2.5_ (1)	2.065	0.465	7.883	3.228–19.250	<0.001
Gender (1)	−0.429	0.464	0.651	0.262–1.616	0.355
Age	0.019	0.455	1.019	0.418–2.483	0.968
Mother's education (1)	−0.786	0.642	0.456	0.130–1.602	0.221
Mother's occupation (1)	−1.159	0.790	0.314	0.067–1.477	0.143
Constant	0.279	0.852	1.322		0.743

### 3.8. Multiple binary logistic regression analysis: model diagnostics

Following the multiple binary logistic regression analysis presented in Table 6, model diagnostics were performed to assess the goodness-of-fit and validity of the final model. The **Hosmer-Lemeshow (HL) test** was conducted to evaluate the agreement between the observed and predicted outcomes. The test yielded a non-significant result (χ² = 4.643, df = 8, p = 0.795), indicating **a good fit of the model to the data.** This suggests that the **model's predictions** are **consistent** with the observed prevalence of RTI across different risk groups.

Furthermore, **residual plots** were examined to identify any potential issues with the model's assumptions, such as outliers or nonlinear patterns. **No significant patterns** or **influential outliers** were detected, providing **additional confidence in the robustness of the model** and **the reliability of the estimated odds ratios**. These diagnostic checks affirm that the multivariate model provides **a valid and appropriate representation** of the relationships between the predictors and the outcome in this dataset, and the full outputs can be found in Table S2 of the Supplementary Materials.

## 4. Discussion

This study found that proximity to areas with poor air quality (high PM_2.5_ levels) is significantly associated with an increased incidence of respiratory disorders in children (χ² = 22.154, p < 0.001). The calculated Phi coefficient (**φ** = 0.475) indicates a **moderate to large association** between PM_2.5_ exposure and respiratory tract infection status. These findings directly highlight the potential burden of air pollution, especially in vulnerable populations such as children living in polluted urban environments like Jakarta.

The significantly higher prevalence of RTI in the high PM_2.5_ exposure group (71.43%) compared to the low exposure group (25.86%) underscores the substantial impact of air pollution on the respiratory health of these children. The odds ratio of 7.167 (95% CI: 3.050–16.837) further suggests that children in high PM_2.5_ exposure areas have approximately 7.167 times higher odds of experiencing RTI compared to their counterparts in low exposure areas, reinforcing the significant association observed. While statistical analysis did not reveal a significant difference in the proportion of maternal education levels between the high and low PM_2.5_ exposure groups, the high prevalence of low maternal education in both groups suggests that associated socioeconomic factors may have contributed to the overall high rates of RTI observed in both exposure groups. Future research should consider controlling for these socioeconomic factors to better understand the independent effect of PM_2.5_ exposure.

To further explore potential confounding factors, we examined the relationship between maternal socioeconomic status, as indicated by maternal education and occupation, with both PM_2.5_ exposure levels and the occurrence of RTI. Chi-square analysis revealed no statistically significant association between maternal education level (low vs. medium) and the PM_2.5_ exposure group [χ²(1) = 0.045, p = 0.833]. Similarly, no significant association was found between maternal occupation (blue collar vs. semi-professional) and the PM_2.5_ exposure group [χ²(1) = 0.006, p = 0.937]. Furthermore, Chi-square tests also indicated no statistically significant associations between maternal education level and the occurrence of RTI [χ²(1) = 0.233, p = 0.629], nor between maternal occupation and RTI [χ²(1) = 0.447, p = 0.504] in our study population. While these findings suggest that, within our sample, maternal education and occupation as categorized were not directly linked to either PM_2.5_ exposure or RTI occurrence at a statistically significant level, the overall predominance of mothers with low levels of education in our study population, as discussed earlier, may still represent an underlying socioeconomic context influencing the general health vulnerability of the children.

The findings of this study demonstrate a significant association between PM_2.5_ exposure levels and the occurrence of RTIs in elementary school children. In addition to PM_2.5_ exposure, other factors may have contributed to the high prevalence of RTI observed in this research. The socioeconomic characteristics of the study population, marked by a dominant proportion of mothers with low levels of education, likely played a significant role. Low maternal education is often correlated with limited health literacy, less adequate housing conditions, restricted access to healthcare services, and suboptimal nutritional status [44],[45]. These factors can increase children's susceptibility to infections, including RTI, irrespective of PM_2.5_ exposure levels [46].

While statistical analysis did not reveal a significant difference in the proportion of maternal education levels between the high and low PM_2.5_ exposure groups, it is important to note that our study used maternal education and occupation as proxies for socioeconomic status. These variables do not fully capture the complexity of SES, such as household income, or housing quality, and the high prevalence of low maternal education in both groups suggests that associated socioeconomic factors may still have contributed to the elevated RTI occurrence observed. Therefore, the observed lack of association between SES and RTI may reflect measurement limitations rather than a true absence of effect. Consequently, the implications for public health policy should be interpreted cautiously, as our study provides preliminary evidence rather than definitive guidance for specific interventions. Future research with more comprehensive SES measures is needed to better understand the independent effects of PM_2.5_ exposure and to inform policy decisions.

These results are in line with previous studies showing that PM_2.5_ particles can enter the respiratory tract and cause irritation and inflammation [1],[2],[6],[47]. Chronic exposure to high levels of PM_2.5_ has also been linked to experiencing more frequent respiratory tract infections and potentially decreased lung function, which may impact their health in adulthood [6],[48].

Our findings are consistent with studies conducted in other urban settings with high air pollution levels. For instance, research in Zhejiang Province, China [17], India [7], and Los Angeles [49] has also reported a higher prevalence of respiratory symptoms and infections among children residing in areas with elevated PM_2.5_ concentrations. The consistency of these findings across geographically diverse locations strengthens the evidence for the detrimental effects of PM_2.5_ on children's respiratory health.

However, this study uniquely contributes to the existing literature by specifically examining this association within the context of Jakarta, Indonesia, a densely populated urban environment with known high levels of air pollution, which has not been extensively studied in this regard. A key strength and novelty of this study is the development and rigorous validation of the RAAEC-C instrument for RTI assessment, as no standardized instrument is currently available for RTI diagnosis due to PM_2.5_ exposure. Our comprehensive validation process, which included expert review for content validity and a reliability assessment using Cohen's Kappa and the intraclass correlation coefficient (ICC), ensures the consistency and credibility of our measurements. The specific pollutants and exposure levels may vary across these studies, potentially contributing to variations in the detrimental effects observed.

Biologically, PM_2.5_ particles, due to their small size, can penetrate deep into lung tissue and reach the alveoli, where they can trigger inflammatory responses and oxidative stress [3],[50]. In children, whose respiratory systems are still developing and whose immune systems are immature, these inflammatory processes can be particularly damaging and may impair their ability to effectively combat respiratory tract infections, thus increasing their susceptibility to RTI [51],[52]. The present study's findings are consistent with this biological plausibility.

To examine potential differences in RTI incidence between genders in the overall study population, a Chi-square test was conducted comparing the occurrence of RTI in males and females. The results of this analysis (Pearson Chi-square = 0.000, df = 1, p = 0.928) indicated no statistically significant association between gender and the presence of RTI in the total sample.

Further analysis explored the relationship between PM_2.5_ exposure level and RTI incidence within specific gender subgroups. Among males, a statistically significant association was observed, with a higher proportion of RTIs reported in the high PM_2.5_ exposure group compared to the low exposure group [χ²(1) = 10.873, p = 0.001]. Similarly, a significant association was also found in the female subgroup, with a greater incidence of RTI among those exposed to higher levels of PM_2.5_ [χ²(1) = 11.755, p = 0.001]. These findings suggest that the detrimental effects of PM_2.5_ on respiratory health, leading to increased susceptibility to RTI, are evident in both male and female children within this study population.

Although multivariate binary logistic regression analysis adjusting for confounders (such as age, gender, maternal education, and maternal occupation) confirmed PM_2.5_ exposure as a strong independent predictor of RTI, the relatively small sample size and limited event counts could compromise model stability. To address this concern, sensitivity analyses were conducted using models with a reduced set of covariates and models including only PM_2.5_ exposure. These analyses showed results consistent in both direction and magnitude with the main model, suggesting that the observed associations are robust. Still, the findings from the multivariate analysis should be interpreted with caution. Future studies with larger sample sizes and more robust adjustments for confounding factors are necessary to validate these associations and further clarify the role of PM_2.5_ exposure in respiratory health outcomes.

It should be emphasized that the cross-sectional design of this study limits our ability to establish causality. The observed associations between PM_2.5_ exposure and RTIs should therefore be interpreted as indicative of a relationship rather than a causal effect. Longitudinal studies will be necessary in the future to confirm temporal and causal links.

The results of this study are consistent with the findings of other studies conducted in various countries. For example, studies in larger cities such as Beijing [53] and Mexico City [54] also report an increase in respiratory morbidities in children living in areas of high air pollution. This study adds new evidence showing the impact of PM_2.5_ on respiratory illnesses, such as respiratory tract infections (RTIs), in the Indonesian context, a region where such data is crucial for informing public health strategies. To the best of our knowledge, this study is among the first to specifically quantify the association between long-term PM_2.5_ exposure and the prevalence of RTI in elementary school children within the Jakarta metropolitan area.

More recent studies further corroborate these findings in pediatric populations. Comotti et al. (2025) reported that exposure to ambient air pollution significantly increased hospitalization risk in infants with bronchiolitis [55]. Similarly, Milani et al. (2022) demonstrated that short-term exposure to PM_2.5_ and PM_10_ was associated with greater bronchiolitis severity, particularly in RSV-related cases [56]. Brusselen et al. (2024) also found that both short- and medium-term exposure to particulate matter and NO₂ were linked to increased bronchiolitis risk in infants [57]. Finally, a systematic review and meta-analysis by Carugno et al. (2018) confirmed consistent associations between PM_2.5_, PM_10_, and NO₂ exposure and increased bronchiolitis hospitalizations across multiple studies [58].

Taken together, this emerging body of evidence strengthens the biological plausibility and external validity of our findings, suggesting that air pollution—particularly PM_2.5_—plays a major role in the burden of pediatric respiratory tract infections.

This research also considers that various other factors can influence children's respiratory health, such as nutritional status, exposure to cigarette smoke, and indoor environmental conditions. The methods of this study attempted to minimize the influence of some of these confounders through the selection of demographically and environmentally similar school communities and the use of inclusion/exclusion criteria. The significant association observed despite these efforts further highlights the contribution of air pollution as a primary preventable risk factor for respiratory tract infections in children.

### 4.1. Public health implications

These findings have important implications for public health policy. Reducing emissions of air pollutants, especially from motor vehicles and industry, should be considered a priority area to protect children's health. In addition, increasing public awareness about the dangers of PM_2.5_ and preventive measures, such as the use of masks and air purifiers, is also important. Implementing policies that support green spaces in urban areas can help reduce PM_2.5_ concentrations and provide a healthier environment for children. Given the significant association found in this study, targeted interventions in high PM_2.5_ exposure areas are urgently needed to mitigate the increased risk of RTI in children. Our findings suggest the importance of considering air quality considerations in urban planning and public health initiatives in Jakarta and potentially other similar urban environments in Indonesia.

### 4.2. Generalizability of the findings

In interpreting these findings, it is important to consider the generalizability of the study results. The study population consisted of elementary school children residing in urban areas of Jakarta and Bandung, where air pollution levels are notably high. Therefore, the findings may not be fully applicable to children living in rural regions, areas with lower pollution levels, or different socio-economic environments. Variations in access to healthcare, housing conditions, nutritional status, and other environmental or lifestyle factors across different settings could influence the observed associations. Thus, the results are most generalizable to urban children exposed to high levels of air pollution in Indonesia or similar environments, and caution should be exercised when extrapolating these findings to other populations.

### 4.3. Study limitations

This study has several limitations that need to be noted.

**First**, PM_2.5_ exposure measurements were based on air quality monitoring data at the area level, which may not fully represent individual exposure levels experienced by each child, as children move between multiple microenvironments (home, school, transport) with varying pollution levels. Although personal exposure monitoring or time-weighted exposure models could provide more precise estimates, these approaches were not feasible in the current study due to the large geographic area, logistical constraints, limited resources, and the high cost and time required for individual-level monitoring. This limitation may have introduced non-differential misclassification, likely biasing the observed associations toward the null, and consequently preventing causal inference regarding PM_2.5_ and RTI outcomes. Additionally, the study compared two cities that differ in altitude, climate, socioeconomic composition, healthcare access, and potential exposure to other air pollutants. While we attempted to minimize confounding variables by selecting schools with similar socioeconomic backgrounds and applying strict inclusion/exclusion criteria, residual confounding from these factors cannot be fully excluded. The exposure assessment was also limited to PM_2.5_ measurements at study sites and did not capture temporal variability, seasonal changes, or co-pollutant concentrations such as NO₂, SO₂, or ultrafine particles. Therefore, the observed associations cannot be attributed solely to PM_2.5_.

**Second**, the cross-sectional design of this study limits our ability to establish a causal relationship between PM_2.5_ exposure and the incidence of respiratory tract infections (RTIs). While data collection at both sites was conducted in the same season to minimize temporal variation, differences in viral circulation between areas could still have influenced the observed prevalence. However, the substantially higher RTI prevalence in Jakarta, despite climatic conditions in Pangalengan being more favorable for viral persistence, suggests that the large difference in PM_2.5_ exposure is a more plausible driver of the observed association. This finding mitigates the limitation of the study design. Longitudinal studies with virological surveillance are needed to confirm these findings.

**Third**, the study included 107 participants, which limits the statistical power of the multivariate models and may result in overfitting. Consequently, the study has limited ability to detect interaction effects or to adjust simultaneously for multiple confounders. Although the logistic regression model met the minimal 10 events per variable rule, this limitation should be considered when interpreting the findings, as it may increase the risk of overestimating the odds ratios. It is important to note that this large OR of 7.167 likely reflects the influence of unmeasured confounding factors. This overestimation is also likely due to the relatively high prevalence of RTI in the study population. Despite these limitations, model diagnostics, including the Hosmer–Lemeshow goodness-of-fit test and residual plots, indicated adequate model fit and no influential outliers. In addition, sensitivity analyses confirmed that the main results remained consistent, supporting the robustness of the reported associations.

**Fourth**, although nutritional status and immunization history were controlled through inclusion criteria (only children with normal BMI-for-age and complete immunization were enrolled), and maternal education and occupation were included in the multivariate model, several important confounders could not be assessed. These include household income, indoor air quality, prior RTI history, long-term PM_2.5_ exposure, lifestyle behaviors (e.g., diet, physical activity), and genetic predispositions. Consequently, the observed associations should be interpreted with caution, as residual confounding may have inflated the effect estimates. In addition, the odds ratio may be overestimated due to the relatively high prevalence of RTIs in the study population.

Finally, future studies should consider incorporating a wider range of socio-economic, environmental, and behavioral variables and exploring potential modifying factors such as genetic susceptibility, which may influence children's vulnerability to air pollution. Furthermore, future research should aim to utilize individual-level PM_2.5_ exposure data and employ longitudinal study designs to establish temporal relationships and confirm the causal link between PM_2.5_ exposure and the incidence and severity of respiratory tract infections in children in Indonesia.

## 5. Conclusions

This cross-sectional study identified a significant association between ambient PM_2.5_ exposure levels and an increased occurrence of respiratory tract infections (RTIs) in school-aged children. Using PM_2.5_ concentration measurements and health data collected through interviews and physical examinations in Jakarta and Bandung, we found that children in areas with high PM_2.5_ levels had a significantly higher prevalence of RTI and approximately 7.167 times higher odds of experiencing RTIs (OR = 7.167, p < 0.001) compared to children in low PM_2.5_ exposure areas. These findings highlight a strong association and underscore the potential health burden of air pollution in urban environments such as Jakarta, but they should be interpreted as preliminary evidence rather than definitive proof of causality.

Additionally, although socioeconomic background factors such as maternal education and employment were explored, no statistically significant associations were found between these variables and either PM_2.5_ exposure or RTI occurrence. This null finding should be interpreted with caution, since maternal education and occupation were only proxy indicators of socioeconomic status and may not capture the full complexity of SES.

This research highlights that exposure to high PM_2.5_ levels poses a significant risk for respiratory health in children. However, the cross-sectional nature of this study limits causal inference. Although several potential confounders were considered in the study design, residual confounding cannot be excluded. Given the significant association observed, the evidence provides preliminary support for policies aimed at reducing children's exposure to air pollution. Further longitudinal research is needed to better understand the long-term impacts of PM_2.5_ exposure, to further investigate potential causal pathways, and to develop more effective interventions.

Efforts to control emissions from transportation and industrial sources in urban areas may play an important role in safeguarding children's health. Special protection and targeted interventions for children residing in areas with high levels of air pollution should be considered. While the findings are most applicable to children in urban areas with high pollution levels, further research is needed to confirm their generalizability to different populations.

The implications of these findings suggest that policy measures aimed at reducing air pollutant emissions and increasing public awareness about the dangers of PM_2.5_, especially for vulnerable populations like children in Jakarta, should be considered. Moreover, measures such as controlling pollution sources and promoting protective behaviors may play an important role. Further longitudinal research is needed to better understand the long-term impacts of PM_2.5_ exposure and to develop more effective interventions. Special protection and targeted interventions for children residing in areas with high levels of air pollution are paramount.

Overall, this study provides important evidence highlighting the significant association between PM_2.5_ exposure and increased odds of respiratory tract infections in school-aged children in Jakarta, underscoring the urgency for public health policies and environmental actions to protect children's respiratory health.

Furthermore, subgroup analyses revealed a significant association between higher PM_2.5_ exposure and increased RTI incidence in both male and female children. This underscores the widespread impact of PM_2.5_ on respiratory health in this vulnerable population, affecting both genders significantly. Given the cross-sectional design, causal inference is limited. Nevertheless, the consistent evidence across the overall sample and both gender subgroups reinforces the urgency for policy interventions to reduce air pollution, promote green infrastructure, and raise public awareness, aiming to protect the respiratory health of all children in highly polluted urban settings.

Although potential confounders such as nutritional status, household smoking exposure, and environmental conditions were considered, PM_2.5_ exposure remained a key independent risk factor. While no cases of asthma were reported, the findings highlight the vulnerability of children's respiratory health to air pollution.

While the primary focus of this study was the impact of PM_2.5_, we also examined the potential role of maternal socioeconomic factors, specifically education and occupation, on both PM_2.5_ exposure and RTI occurrence. Our analysis revealed no statistically significant associations between maternal education level (low vs. medium) and PM_2.5_ exposure group, nor between maternal occupation (blue collar vs. semi-professional) and PM_2.5_ exposure group. Similarly, no significant associations were found between maternal education level and RTI or between maternal occupation and RTI within our study population. However, it is important to acknowledge the observed high prevalence of mothers with low levels of education in our sample, suggesting a potentially relevant broader socioeconomic context that may influence children's overall health vulnerability. Future research should further investigate the complex interplay between environmental exposures and socioeconomic determinants in shaping respiratory health outcomes in this setting.

Overall, this study provides important insights to guide public health policies and environmental actions to protect children's respiratory health. These findings provide preliminary evidence that may inform actions to protect the respiratory health of children in Jakarta and other cities facing similar air pollution challenges. However, given its cross-sectional and between-city comparison design, this study should be regarded as hypothesis-generating rather than confirmatory, underscoring the need for future longitudinal and within-city gradient studies to strengthen causal inference.

### 5.1. Future directions

#### 5.1.1 Future research

To strengthen the evidence base and establish causal relationships, future studies should utilize **prospective cohort designs** to track children's respiratory health outcomes over time in relation to PM_2.5_ exposure. Additionally, research should explore the potential modifying effects of **socioeconomic status, nutritional factors, household environmental conditions**, and **genetic susceptibility** on the relationship between air pollution and respiratory tract infections.

Future studies incorporating within-city exposure gradient designs would strengthen causal inference compared to the between-city approach used here.

#### 5.1.2 Mitigation and intervention strategies

Further studies are needed to evaluate the **effectiveness of mitigation interventions**, such as the use of **air purifiers in schools**, **promotion of mask-wearing during high pollution episodes**, and **community education programs** aimed at increasing awareness of the health risks associated with PM_2.5_ exposure. In addition, **policy-focused research** is necessary to assess the impact of **urban planning initiatives** (e.g., creation of green spaces, traffic control measures) on reducing children's exposure to air pollutants and improving respiratory health outcomes.

## Use of AI tools declaration

The authors declare they have not used Artificial Intelligence (AI) tools in the creation of this article.

## Publication ethics statement

This study complies with the ethical publishing standards. Author affirms that the manuscript is original, has not been published previously, and is not under consideration by another journal. The authors have adhered to ethical research and publication guidelines, including accurate reporting of data and acknowledgement of all contributors.

## Research ethics statement

This research has been conducted with full adherence to the ethical principles relevant to the field of study. Every stage of the research, from planning, data collection, analysis, to the preparation of the report, has been carried out with due consideration for the rights and interests of all parties involved.

We ensured that all research participants provided informed consent voluntarily, without coercion, and were fully informed about the purpose, methods, and potential risks of the study. All data obtained has been kept confidential using strict security procedures and will only be used for the purposes outlined in the research.

This study also avoids any form of plagiarism and properly acknowledges all sources in accordance with academic standards. We are committed to maintaining scientific integrity and ensuring that all research findings are presented objectively and transparently.

## Funding

This research article was conducted independently by the authors without any external funding.


