# Supplementary material for: The association between PM2.5 level and respiratory tract infections among children: A cross-sectional study
Source: AIMS Public Health. 2025 Nov 18;12(4):1084–114. doi: 10.3934/publichealth.2025055 (PMC12667370; doi:10.3934/publichealth.2025055)
Supplement: Supplementary file 1 [file publichealth-12-04-055-s001.pdf]

Public Health

AIMS Public Health, 12(4): 1084–1114.
DOI: 10.3934/publichealth.2025055
Received: 22 July 2025
Revised: 27 September 2025
Accepted: 14 October 2025
Published: 18 November 2025

http://www.aimspress.com/journal/aimsph

Research article

The association between $PM_{2.5}$ level and respiratory tract infections among children: A cross-sectional study

Hari Krismanuel* **and Purnamawati Tjhin**

Faculty of Medicine, Universitas Trisakti, Jakarta, Indonesia

* **Correspondence:** Email: hari_krismanuel@trisakti.ac.id.

Supplementary

Table S1. Completed STROBE Checklist for a Cross-Sectional Study on $PM_{2.5}$ and Respiratory Tract Infections in Children.

	Item No.	Recommendation	Reported on page #
**Title and abstract**	1	(a) Indicate the study's design with a commonly used term in the title or the abstract.	1
		(b) Provide in the abstract an informative and balanced summary of what was done and what was found.	1
**Introduction**			
Background and rationale	2	Explain the scientific background and rationale for the investigation being reported.	2–3
Objectives	3	State specific objectives, including any prespecified hypotheses.	2–3
**Methods**			
Study design	4	Present key elements of study design early in the paper.	3–4
Setting	5	Describe the setting, locations, and relevant dates, including periods of recruitment, data collection, and follow-up.	4–8
Participants	6	(a) Give the eligibility criteria, and the sources and methods of selection of participants.	4–8
		(b) For matched studies, give matching criteria and number of exposed and unexposed.	N/A
Variables	7	Clearly defined all outcomes, exposures, predictors, potential confounders, and effect modifiers. Give diagnostic criteria, if applicable.	6–11
Data sources/ measurement	8*	For each variable of interest, give sources of data and detail of methods of assesment (measurements). Describe comparability of assessment methods if there is more than one group.	7–11
Bias	9	Describe any efforts to address potential sources of bias.	9–10
Study size	10	Explain how the study size was arrived at.	6–7
Quantitative variables	11	Explained how quantitative variables were handled in the analyses. If applicable, describe which groupings were chosen and why.	9–10
Statistical methods	12	(a) Describe all statistical methods, including those used to control for confounding.	9–10
		(b) Describe any methods used to examined subgroups and interactions.	9–10
		(c) Explain how missing data were addressed.	8
		(d) If applicable, explain how loss to follow-up was addressed for each outcome.	8

		(e) Describe any sensitivity analyses.	10
**Results**			
Participants	13*	(a) Report numbers of individuals at each stage of study-e.g., numbers potentially eligible, examined for eligibility, confirmed eligible, included in the study, completing follow-up, and analysed.	4–5
		(b) Give reasons for non participation at each stage.	5, 11
		(c) Consider use of a flow diagram.	N/A
Descriptive data	14*	(a) Give characteristics of study participants (e.g., demographic, clinical, social) and information on exposures and potential confounders.	14
		(b) Indicate number of participants with missing data for each variable on interest.	10–11
Outcome data	15*	Report numbers of outcomes event or summary measures.	11–13
	16	(a) Give unadjusted estimates and, if applicable, confounder-adjusted estimates and their precision (eg., 95% Confidence Interval). Make clear which confounders were adjusted for and why they were included.	12–13
		(b) Reports category boundaries when continuous variables were categorized.	11–12
		(c) If relevant, consider translating estimates of relative risk into absolute risk for a meaningful time period.	N/A
Other analyses	17	Report other analyses done-eg., analyses of subgroups and interactions, and sensitivity analyses.	9, 13–15
**Discussion**			
Key results	18	Summarize key results with reference to study objectives.	16–18
Limitations	19	Discuss limitations of of the study, taking into account sources of potential bias or imprecision? Discuss both direction and magnitude of any potential bias.	19
Interpretations	20	Give a cautious overall interpretations of results considering objectives, limitations, multiplicity of analyses, results from similar studies, and other relevant evidence.	16–21
Generalisability	21	Discuss the generalisability (external validity) of the study results.	19–20
**Other information**			
Funding	22	Give the source of funding and the role of funders for the present study and, if applicable, for the original study on which the present article is based.	N/A

Note: This file is an author-completed version of the official STROBE Checklist for cross-sectional studies. The STROBE Initiative retains the original copyright.

Table S2. Statistical analysis results.

S2.1. Chi-Square test of independence results for age group distribution between $PM_{2.5}$ exposure groups.

Crosstabs

Case Processing Summary

	Cases					
	Valid		Missing		Total	
	N	Percent	N	Percent	N	Percent
age × $PM_{2.5}$	107	100.0%	0	0.0%	107	100.0%

age * $PM_{2.5}$ Crosstabulation

			$PM_{2.5}$		Total
			low	high	
age	6–9 tahun	Count	22	20	42
		% within age	52.4%	47.6%	100.0%
		% within $PM_{2.5}$	37.9%	40.8%	39.3%
	10–12 tahun	Count	36	29	65
		% within age	55.4%	44.6%	100.0%
		% within $PM_{2.5}$	62.1%	59.2%	60.7%
Total		Count	58	49	107
		% within age	54.2%	45.8%	100.0%
		% within $PM_{2.5}$	100.0%	100.0%	100.0%

Chi-Square Tests					
	Value	df	Asymptotic Significance (2-sided)	Exact Sig. (2-sided)	Exact Sig. (1-sided)
Pearson Chi-Square	0.093[a]	1	0.761		
Continuity Correction[b]	0.011	1	0.916		
Likelihood Ratio	0.093	1	0.761		
Fisher's Exact Test				0.843	0.457
Linear-by-Linear Association	0.092	1	0.762		
N of Valid Cases	107				

Note: [a]0 cells (0%) have expected count less than 5. The minimum expected count is 19, 23; [b]Computed only for a 2 × 2 table.

S2.2. Chi-Square Test of Independence Results for Gender Group Distribution between $PM_{2.5}$ Exposure Groups.

Crosstabs

Case Processing Summary						
	Cases					
	Valid		Missing		Total	
	N	Percent	N	Percent	N	Percent
gender × $PM_{2.5}$	107	100.0%	0	0.0%	107	100.0%

**gender * $PM_{2.5}$ Crosstabulation**					
			$PM_{2.5}$		Total
			low	high	
gender	male	Count	26	28	54
		% within gender	48.1%	51.9%	100.0%
		% within $PM_{2.5}$	44.8%	57.1%	50.5%
	female	Count	32	21	53
		% within gender	60.4%	39.6%	100.0%
		% within $PM_{2.5}$	55.2%	42.9%	49.5%
Total		Count	58	49	107
		% within gender	54.2%	45.8%	100.0%
		% within $PM_{2.5}$	100.0%	100.0%	100.0%

Chi-Square Tests					
	Value	df	Asymptotic Significance (2-sided)	Exact Sig. (2-sided)	Exact Sig. (1-sided)
Pearson Chi-Square	1.611[a]	1	0.204		
Continuity Correction[b]	1.156	1	0.282		
Likelihood Ratio	1.616	1	0.204		
Fisher's Exact Test				0.246	0.141
Linear-by-Linear Association	1.596	1	0.206		
N of Valid Cases	107				

Note: [a]0 cells (0%) have expected count less than 5. The minimum expected count is 24,27; [b]Computed only for a 2 × 2 table.

S2.3. Chi-Square Test of Independence between $PM_{2.5}$ Exposure and Respiratory Tract Infections (RTI).

Case Processing Summary						
	Cases					
	Valid		Missing		Total	
	N	Percent	N	Percent	N	Percent
$PM_{2.5}$ × RTI	107	100.0%	0	0.0%	107	100.0%

$PM_{2.5}$ * RTI Crosstabulation					
			RTI		Total
			no	yes	
$PM_{2.5}$	low	Count	43	15	58
		% within $PM_{2.5}$	74.1%	25.9%	100.0%
		% within RTI	75.4%	30.0%	54.2%
	high	Count	14	35	49
		% within $PM_{2.5}$	28.6%	71.4%	100.0%
		% within RTI	24.6%	70.0%	45.8%
Total		Count	57	50	107
		% within $PM_{2.5}$	53.3%	46.7%	100.0%
		% within RTI	100.0%	100.0%	100.0%

Chi-Square Tests	Value	df	Asymptotic Significance (2-sided)	Exact Sig. (2-sided)	Exact Sig. (1-sided)
Pearson Chi-Square	22.154[a]	1	0.000		
Continuity Correction[b]	20.361	1	0.000		
Likelihood Ratio	22.938	1	0.000		
Fisher's Exact Test				0.000	0.000
Linear-by-Linear Association	21.947	1	0.000		
N of Valid Cases	107				

Note: [a]0 cells (0%) have expected count less than 5. The minimum expected count is 22,90; [b]Computed only for a 2 × 2 table.

S2.4. Chi-Square Test of Independence between Gender and Respiratory Tract Infections (RTI).

Crosstabs

Case Processing Summary						
	Cases					
	Valid		Missing		Total	
	N	Percent	N	Percent	N	Percent
gender × RTI	107	100.0%	0	0.0%	107	100.0%

Gender × RTI Crosstabulation					
			RTI		Total
			no	yes	
gender	male	Count	29	25	54
		% within gender	53.7%	46.3%	100.0%
		% within RTI	50.9%	50.0%	50.5%
	female	Count	28	25	53
		% within gender	52.8%	47.2%	100.0%
		% within RTI	49.1%	50.0%	49.5%

Total	Count	57	50	107
	% within gender	53.3%	46.7%	100.0%
	% within RTI	100.0%	100.0%	100.0%

Chi-Square Tests

	Value	df	Asymptotic Significance (2-sided)	Exact Sig. (2-sided)	Exact Sig. (1-sided)
Pearson Chi-Square	0.008[a]	1	0.928		
Continuity Correction[b]	0.000	1	1000		
Likelihood Ratio	0.008	1	0.928		
Fisher's Exact Test				1.000	0.541
Linear-by-Linear Association	0.008	1	0.928		
N of Valid Cases	107				

Note: [a]0 cells (0%) have expected count less than 5. The minimum expected count is 24.77; [b]Computed only for a 2 × 2 table.

S2.5. Chi-Square Test of Association between Age (Categorized) and Respiratory Tract Infections (RTI).

Crosstabs

Case Processing Summary

	Cases					
	Valid		Missing		Total	
	N	Percent	N	Percent	N	Percent
age × RTI	107	100.0%	0	0.0%	107	100.0%

age × RTI Crosstabulation

			RTI		Total
			no	yes	
age	6.00	Count	0	1	1
		% within age	0.0%	100.0%	100.0%
		% within RTI	0.0%	2.0%	0.9%
	7.00	Count	6	2	8
		% within age	75.0%	25.0%	100.0%
		% within RTI	10.5%	4.0%	7.5%
	8.00	Count	5	9	14
		% within age	35.7%	64.3%	100.0%
		% within RTI	8.8%	18.0%	13.1%
	9.00	Count	11	8	19
		% within age	57.9%	42.1%	100.0%
		% within RTI	19.3%	16.00%	17.8%
	10.00	Count	11	7	18
		% within age	61.1%	38.9%	100.0%
		% within RTI	19.3%	14.0%	16.8%
	11.00	Count	10	8	18
		% within age	55.6%	44.4%	100.0%
		% within RTI	17.5%	16.0%	16.8%
	12.00	Count	14	15	29
		% within age	48.3%	51.7%	100.0%
		% within RTI	24.6%	30.0%	27.1%
Total		Count	57	50	107
		% within age	53.3%	46.7%	100.0%

	% within RTI	100.0%	100.0%	100.0%

Chi-Square Tests

	Value	df	Asymptotic Significance (2-sided)
Pearson Chi-Square	5.327[a]	6	0.503
Likelihood Ratio	5.809	6	0.445
Linear-by-Linear Association	0.031	1	0.859
N of Valid Cases	107		

Note: [a]4 cells (28,6%) have expected count less than 5. The minimum expected count is 47.

S2.6. Detailed SPSS Output for Chi-Square Test of Association between $PM_{2.5}$ Exposure and RTI in the Male Subgroup (n = 54).

Crosstabs

Case Processing Summary

	Cases					
	Valid		Missing		Total	
	N	Percent	N	Percent	N	Percent
$PM_{2.5}$ × RTI	54	100.0%	0	0.0%	54	100.0%

$PM_{2.5}$ × RTI Crosstabulation

			RTI		Total
			no	yes	
$PM_{2.5}$	male	Count	20	6	26
		% within gender	76.9%	23.1%	100.0%
		% within RTI	69.0%	24.0%	48.1%
	female	Count	9	19	28
		% within gender	32.1%	67.9%	100.0%
		% within RTI	31.0%	76.0%	51.9%
Total		Count	29	25	54
		% within gender	53.7%	46.3%	100.0%
		% within RTI	100.0%	100.0%	100.0%

Chi-Square Tests

	Value	df	Asymptotic Significance (2-sided)	Exact Sig. (2-sided)	Exact Sig. (1-sided)
Pearson Chi-Square	10.873[a]	1	0.001		
Continuity Correction[b]	9.147	1	0.002		
Likelihood Ratio	11.308	1	0.000		
Fisher's Exact Test				0.001	0.001
Linear-by-Linear Association	10.672	1	0.001		
N of Valid Cases	54				

Note: [a]0 cells (0.0%) have expected count less than 5. The minimum expected count is 12.04. [b]Computed only for a 2 × 2 table.

S2.7. Detailed SPSS Output for Chi-Square Test of Association between PM2.5 Exposure and RTI in the Female Subgroup (n = 54).

Crosstabs

Case Processing Summary

	Cases					
	Valid		Missing		Total	
	N	Percent	N	Percent	N	Percent
$PM_{2.5}$ × RTI	53	100.0%	0	0.0%	53	100.0%

$PM_{2.5}$ × RTI Crosstabulation

			RTI		Total
			no	yes	
$PM_{2.5}$	low	Count	23	9	32
		% within $PM_{2.5}$	71.9%	28.1%	100.0%
		% within RTI	82.1%	36.0%	60.4%
	high	Count	5	16	21
		% within $PM_{2.5}$	23.8%	76.2%	100.0%
		% within RTI	17.9%	64.0%	39.6%
Total		Count	28	25	53
		% within $PM_{2.5}$	52.8%	47.2%	100.0%
		% within RTI	100.0%	100.0%	100.0%

Chi-Square Tests

	Value	df	Asymptotic Significance (2-sided)	Exact Sig. (2-sided)	Exact Sig. (1-sided)
Pearson Chi-Square	11.755[a]	1	0.001		
Continuity Correction[b]	9.905	1	0.002		
Likelihood Ratio	12.227	1	0.000		
Fisher's Exact Test				0.001	0.001
Linear-by-Linear Association	11.533	1	0.001		
N of Valid Cases	53				

Note: [a]0 cells (0.0%) have expected count less than 5. The minimum expected count is 9.91. [b]Computed only for a 2 × 2 table.

S2.8. Detailed SPSS Output for Chi-Square Test of Association between mother's education and $PM_{2.5}$.

Crosstabs

Case Processing Summary

	Cases					
	Valid		Missing		Total	
	N	Percent	N	Percent	N	Percent
mother_education × $PM_{2.5}$	107	100.0%	0	0.0%	107	100.0%

**Mother's_education × $PM_{2.5}$ Crosstabulation**					
			$PM_{2.5}$		
			low	high	Total
Mother_education	low	Count	39	32	71
		% within mother's_education	54.9%	45.1%	100.0%
		% within $PM_{2.5}$	67.2%	65.3%	66.4%
	high	Count	19	17	36
		% within mother's_education	52.8%	47.2%	100.0%
		% within $PM_{2.5}$	32.8%	34.7%	33.6%
Total		Count	58	49	107
		% within mother's_education	54.2%	47.2%	100.0%
		% within $PM_{2.5}$	100.0%	100.0%	100.0%

**Chi-Square Tests**					
	Value	df	Asymptotic Significance (2-sided)	Exact Sig. (2-sided)	Exact Sig. (1-sided)
Pearson Chi-Square	0.045[a]	1	0.833		
Continuity Correction[b]	0.000	1	0.995		
Likelihood Ratio	0.045	1	0.833		
Fisher's Exact Test				0.840	0.497
Linear-by-Linear Association	0.044	1	0.834		
N of Valid Cases	107				

Note: [a]0 cells (0.0%) have expected count less than 5. The minimum expected count is 16.49. [b]Computed only for a 2 × 2 table.

S2.9. Detailed SPSS Output for Chi-Square Test of Association between mother's occupation and $PM_{2.5}$.

Crosstabs

**Case Processing Summary**						
	Cases					
	Valid		Missing		Total	
	N	Percent	N	Percent	N	Percent
mother_occupation × $PM_{2.5}$	107	100.0%	0	0.0%	107	100.0%

**Mother's_occupation × $PM_{2.5}$ Crosstabulation**					
			$PM_{2.5}$		
			low	high	Total
Mother_occupation	low	Count	47	40	87
		% within mother's_occupation	54.0%	46.0%	100.0%
		% within $PM_{2.5}$	81.0%	81.6%	81.3%
	high	Count	11	9	20
		% within mother's_occupation	55.0%	45.0%	100.0%
		% within $PM_{2.5}$	19.0%	18.4%	18.7%
Total		Count	58	49	107
		% within mother's_occupation	54.2%	45.8%	100.0%
		% within $PM_{2.5}$	100.0%	100.0%	100.0%

Chi-Square Tests

	Value	df	Asymptotic Significance (2-sided)	Exact Sig. (2-sided)	Exact Sig. (1-sided)
Pearson Chi-Square	0.006[a]	1	0.937		
Continuity Correction[b]	0.000	1	1.000		
Likelihood Ratio	0.006	1	0.937		
Fisher's Exact Test				1.000	0.569
Linear-by-Linear Association	0.006	1	0.937		
N of Valid Cases	107				

Note: [a]0 cells (0.0%) have expected count less than 5. The minimum expected count is 9.16. [b]Computed only for a 2 × 2 table.

S2.10. Detailed SPSS Output for Chi-Square Test of Association between mother's education and RTI.

Crosstabs

Case Processing Summary

	Cases					
	Valid		Missing		Total	
	N	Percent	N	Percent	N	Percent
mother_education × RTI	107	100.0%	0	0.0%	107	100.0%

Mother's_education × RTI Crosstabulation

			RTI		
			no	yes	Total
Mother_education	low	Count	39	32	71
		% within mother's_education	54.9%	45.1%	100.0%
		% within RTI	68.4%	64.0%	66.4%
	high	Count	18	18	36
		% within mother's_education	50.0%	50.0%	100.0%
		% within RTI	31.6%	36.0%	33.6%
Total		Count	57	50	107
		% within mother's_education	53.3%	46.7%	100.0%
		% within RTI	100.0%	100.0%	100.0%

Chi-Square Tests

	Value	df	Asymptotic Significance (2-sided)	Exact Sig. (2-sided)	Exact Sig. (1-sided)
Pearson Chi-Square	0.233[a]	1	0.629		
Continuity Correction[b]	0.077	1	0.781		
Likelihood Ratio	0.233	1	0.629		
Fisher's Exact Test				0.684	0.390
Linear-by-Linear Association	0.231	1	0.631		
N of Valid Cases	107				

Note: [a]0 cells (0.0%) have expected count less than 5. The minimum expected count is 16.82. [b]Computed only for a 2 × 2 table.

S2.11. Detailed SPSS Output for Chi-Square Test of Association between mother's occupation and RTI.

Crosstabs

Case Processing Summary

	Cases					
	Valid		Missing		Total	
	N	Percent	N	Percent	N	Percent
mother_occupation × RTI	107	100.0%	0	0.0%	107	100.0%

Mother's_occupation × RTI Crosstabulation

			$PM_{2.5}$		
			low	high	Total
Mother_occupation	blue collar	Count	45	42	87
		% within mother's_occupation	51.7%	48.3%	100.0%
		% within RTI	78.9%	84.0%	81.3%
	semi-professional	Count	12	8	20
		% within mother's_occupation	60.0%	40.0%	100.0%
		% within RTI	21.1%	16.0%	18.7%
Total		Count	57	50	107
		% within mother's_occupation	53.3%	46.7%	100.0%
		% within RTI	100.0%	100.0%	100.0%

Chi-Square Tests

	Value	df	Asymptotic Significance (2-sided)	Exact Sig. (2-sided)	Exact Sig. (1-sided)
Pearson Chi-Square	0.447[a]	1	0.504		
Continuity Correction[b]	0.177	1	0.674		
Likelihood Ratio	0.451	1	0.502		
Fisher's Exact Test				0.621	0.339
Linear-by-Linear Association	0.443	1	0.506		
N of Valid Cases	107				

Note: [a]0 cells (0.0%) have expected count less than 5. The minimum expected count is 9.35. [b]Computed only for a 2 × 2 table.

S2.12. Detailed SPSS Output for Crosstabulation of PM2.5 Exposure and Respiratory Tract Infections with Phi and Cramer's V.

Symmetric Measures

		Value	Approximate Significance	Monte Carlo Significance		
				Significance	99% Confidence Interval	
					Lower Bound	Upper Bound
Nominal by Nominal	Phi	0.475	0.000	0.000[c]	0.000	0.000
	Cramer's V	0.475	0.000	0.000[c]	0.000	0.000
N of Valid Cases		107				

Note: [c]Based on 10,000 sampled tables with starting seed 2,000,000.

S2.13. Comparison of Odds Ratio Estimates for Respiratory Tract Infection (RTI) and $PM_{2.5}$ Exposure from Logistic Regression, Risk Estimate, and Mantel-Haenszel Analysis.

Case Processing Summary

Unweighted Cases[a]		N	Percent
Selected Cases	Included in Analysis	107	100.0
	Missing Cases	0	0.0
	Total	107	100.0
Unselected Cases		0	0.0
Total		107	100.0

Note: [a]If weight is in effect, see classification table for the total number of cases.

Dependent Variable Encoding

Original Value	Internal Value
no	0
yes	1

Categorical Variables Codings

		Frequency	Parameter coding (1)
$PM_{2.5}$	low	58	−0.500
	high	49	0.500

Block 0: Beginning Block

Classification Table[a,b]

			Predicted		
			RTI		Percentage Correct
	Observed		no	yes	
Step 0	RTI	no	57	0	100.0
		yes	50	0	0.0
	Overall Percentage				53.3

Note: [a]Constant is included in the model. [b]The cut value is 0.500.

Variables in the Equation

		B	S.E.	Wald	df	Sig.	Exp(B)
Step 0	Constant	−0.131	0.194	0.457	1	0.499	0.877

Variables not in the Equation

			Score	df	Sig.
Step 0	Variables	$PM_{2.5}(1)$	22.154	1	0.000
	Overall Statistics		22.154	1	0.000

Block 1: Method = Enter

Omnibus Tests of Model Coefficients

	Chi-square	df	Sig.

Step 1	Step	22.938	1	0.000
	Block	22.938	1	0.000
	Model	22.938	1	0.000

Model Summary

Step	−2 Log likelihood	Cox & Snell R Square	Nagelkerke R Square
1	124.937[a]	0.193	0.258

Note: [a]Estimation terminated at iteration number 4 because parameter estimates changed by less than 0.001.

Hosmer and Lemeshow Test

Step	Chi-square	df	Sig.
1	0.000	0	

Contingency Table for Hosmer and Lemeshow Test

		RTI = no		RTI = yes		Total
		Observed	Expected	Observed	Expected	
Step 1	1	43	43.000	15	15.000	58
	2	14	14.000	35	35.000	49

Classification Table[a]

			Predicted		
			RTI		Percentage Correct
	Observed		no	yes	
Step 1	RTI	no	43	14	75.4
		yes	15	35	70.0
	Overall Percentage				72.9

Note: [a]The cut value is 0.500.

Variables in the Equation

		B	S.E.	Wald	df	Sig.	Exp(B)	95% CI for EXP(B)	
								Lower	Upper
Step 1[a]	$PM_{2.5}$(1)	1.969	0.436	20.423	1	0.000	7.167	3.050	16.837
	Constant	−0.068	0.218	0.099	1	0.753	0.934		

Note: [a]Variable (s) entered on step 1: $PM_{2.5}$

Chi-Square Tests

	Value	df	Asymptotic Significance (2-sided)	Exact Sig. (2-sided)	Exact Sig. (1-sided)
Pearson Chi-Square	22.154[a]	1	0.000		
Continuity Correction[b]	20.361	1	0.000		
Likelihood Ratio	22.938	1	0.000		
Fisher's Exact Test				0.000	0.000
Linear-by-Linear Association	21.947	1	0.000		
N of Valid Cases	107				

Note: [a]0 cells (0.0%) have expected count less than 5. The minimum expected count is 22.90. [b]Computed only for a 2 × 2 table.

Symmetric Measures

		Value	Asymptotic Standard Error[a]	Approximate T[b]	Approximate Significance
Nominal by Nominal	Phi	0.455			0.000
	Cramer's V	0.455			0.000
Interval by Interval	Pearson's R	0.455	0.086	5.236	0.000[c]
Ordinal by Ordinal	Spearman Correlation	0.455	0.086	5.236	0.000[c]
N of Valid Cases		107			

Note: [a]Not assuming the null hypothesis. [b]Using the asymptotic standard error assuming the null hypothesis. [c]Based on normal approximation.

Risk Estimate

	Value	95% Confidence Interval	
		Lower	Upper
Odds Ratio for $PM_{2.5}$ (low / high)	7.167	3.050	16.837
For cohort RTI = no	2.595	1.625	4.144
For cohort RTI = yes	0.362	0.226	0.580
N of Valid Cases	107		

Tests of Homogeneity of the Odds Ratio

	Chi-Squared	df	Asymptotic Significance (2-sided)
Breslow-Day	0.000	0	
Tarone's	0.000	0	

Tests of Conditional Independence

	Chi-Squared	df	Asymptotic Significance (2-sided)
Cochran's	22.154	1	0.000
Mantel-Haenszel	20.171	1	0.000

Note: Under the conditional independence assumption, Cochran's statistic is asymptotically distributed as a 1 df chi-squared distribution, only if the number of strata is fixed, while the Mantel-Haenszel statistic is always asymptotically distributed as a 1 df chi-squared distribution. Note that the continuity correction is removed from the Mantel-Haenszel statistic when the sum of the differences between the observed and the expected is 0.

Mantel-Haenszel Common Odds Ratio Estimate

Estimate			7.167
Ln (Estimate)			1.969
Standard Error of ln (Estimate)			0.436
Asymptotic Significance (2-sided)			0.000
Asymptotic 95% Confidence Interval	Common Odds Ratio	Lower Bound	3.050
		Upper Bound	16.837
	Ln (Common Odds Ratio)	Lower Bound	1.115
		Upper Bound	2.824

Note: The Mantel-Haenszel common odds ratio estimate is asymptotically normally distributed under the common odds ratio of 1000 assumption. So is the natural log of the estimate.

S2.14. Multiple Binary Logistic Regression Analysis for Confounding Factors Associated with Respiratory Tract Infections (RTIs) among Children, Hosmer-Lemeshow test.

Logistic Regression

Case Processing Summary

Unweighted Cases[a]		N	Percent
Selected Cases	Included in Analysis	107	100.0
	Missing Cases	0	0.0
	Total	107	100.0
Unselected Cases		0	0.0
Total		107	100.0

Note: [a]If weight is in effect, see classification table for the total number of cases.

Dependent Variable Encoding

Original Value	Internal Value
no	0
yes	1

Categorical Variables Codings

		Frequency	Parameter coding (1)
mother_occupation	blue collar	87	−0.500
	semi-professional	20	0.500
gender	male	54	1.000
	female	53	0.000
mother_education	low	71	1.000
	medium	36	0.000
$PM_{2.5}$	low	58	−0.500
	high	49	0.500

Block 0: Beginning Block

Classification Table[a,b]

			Predicted		
			RTI		Percentage Correct
	Observed		no	yes	
Step 0	RTI	no	57	0	100.0
		yes	50	0	0.0
	Overall Percentage				53.3

Note: [a]Constant is included in the model. [b]The cut value is 0.500.

Variables in the Equation

		B	S.E.	Wald	df	Sig.	Exp(B)
Step 0	Constant	−0.131	0.194	0.457	1	0.499	0.877

Variables not in the Equation

			Score	df	Sig.
Step 0	Variables	$PM_{2.5}$ (1)	22.154	1	0.000
		Gender (1)	0.008	1	0.928
		age	0.022	1	0.882
		mother_education (1)	0.233	1	0.629
		mother_occupation (1)	0.447	1	0.504
	Overall Statistics		24.439	5	0.000

Block 1: Method = Enter

**Omnibus Tests of Model Coefficients**				
		Chi-square	df	Sig.
Step 1	Step	25.854	5	0.000
	Block	25.854	5	0.000
	Model	25.854	5	0.000

**Model Summary**			
Step	−2 Log likelihood	Cox & Snell R Square	Nagelkerke R Square
1	122.022[a]	0.215	0.287

Note: [a]Estimation terminated at iteration number 4 because parameter estimates changed by less than 0.001.

**Hosmer and Lemeshow Test**			
Step	Chi-square	df	Sig.
1	4.643	8	0.795

**Contingency Table for Hosmer and Lemeshow Test**						
		RTI = no		RTI = yes		Total
		Observed	Expected	Observed	Expected	
Step 1	1	7	5.736	0	1.264	7
	2	11	11.916	4	3.084	15
	3	5	6.294	3	1.706	8
	4	9	7.913	2	3.087	11
	5	8	7.724	3	3.276	11
	6	5	4.665	4	4.335	9
	7	3	3.664	8	7.336	11
	8	4	3.923	8	8.077	12
	9	3	3.867	12	11.133	15
	10	2	1.299	6	6.701	8

**Classification Table[a]**					
			Predicted		
			RTI		Percentage Correct
	Observed		no	yes	
Step 1	RTI	no	43	14	75.4
		yes	15	35	70.0
	Overall Percentage				72.9

Note: [a]The cut value is 0.500.

Variables in the Equation

		B	S.E.	Wald	df	Sig.	Exp(B)	95% CI for Exp (B)	
								Lower	Upper
Step 1[a]	$PM_{2.5}$(1)	2.065	0.455	20.547	1	0.000	7.883	3.228	19.250
	gender(1)	−0.429	0.464	0.856	1	0.355	0.651	0.262	1.616
	age	0.019	0.455	0.002	1	0.968	1.019	0.418	2.483
	mother_education(1)	−0.786	0.642	1.500	1	0.221	0.456	0.130	1.602
	mother_occupation(1)	−1.159	0.790	2.150	1	0.143	0.314	0.067	1.477
	Constant	0.279	0.852	0.107	1	0.743	1.322		

Note: [a]Variable (s) entered on step 1: $PM_{2.5}$, gender, age, mother_education, mother_occupation.

S2.15. Standardized residuals versus predicted probabilities from Multivariate Binary Logistic Regression Analysis.

```
GRAPH
  /SCATTERPLOT(BIVAR)=PRE_3 WITH ZRE_3
  /MISSING=LISTWISE.
```

Graph

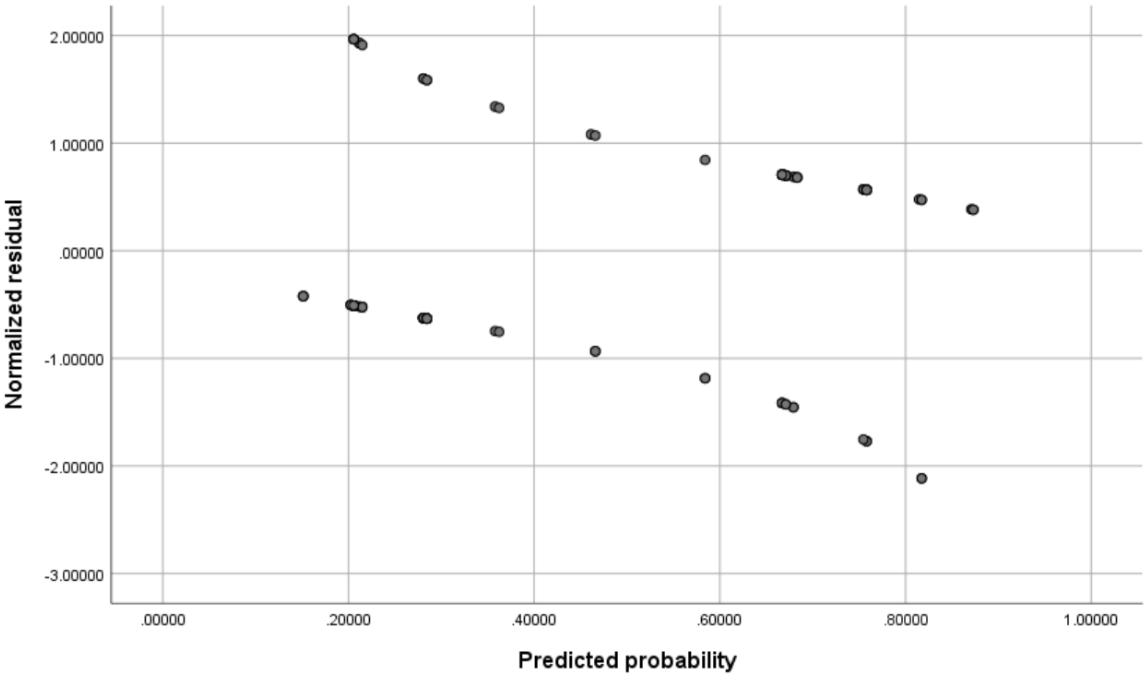

© 2025 the Author(s), licensee AIMS Press. This is an open access article distributed under the terms of the Creative Commons Attribution License (http://creativecommons.org/licenses/by/4.0)
